# Supplementary material for: Human embryonic genome activation initiates at the one-cell stage
Source: Cell Stem Cell. 2022 Feb 3;29(2):209–216.e4. doi: 10.1016/j.stem.2021.11.012 (PMC8826644; doi:10.1016/j.stem.2021.11.012)
Supplement: Document S1. Figures S1 and S2 and Tables S2 and S3 [file mmc1.pdf]

**Cell Stem Cell, Volume *29***

## Supplemental Information

# Human embryonic genome activation

# initiates at the one-cell stage

**Maki Asami, Brian Y.H. Lam, Marcella K. Ma, Kara Rainbow, Stefanie Braun, Matthew D. VerMilyea, Giles S.H. Yeo, and Anthony C.F. Perry**

# SUPPLEMENTAL INFORMATION

## Human embryonic genome activation initiates at the one-cell stage

Maki Asami, Brian Y. H. Lam, Marcella K. Ma, Kara Rainbow, Stefanie Braun, Matthew D. VerMilyea, Giles S. H. Yeo, Anthony C. F. Perry

**This file includes:**

Legends for Figures S1 and S2

Figures S1 and S2

Tables S2 and S3

**Other Supplemental Information for this manuscript includes:**

Table S1 (.xls)

**Figure S1. Human one-cell embryo expression profiles and relationships**

(A) Volcano plot showing genes exhibiting upregulated (up), downregulated (down) and non-changing (nc) expression in one-cell embryos from scRNA-seq. Top upregulated (blue) and down regulated (pink) genes are indicated.

(B) Upregulated gene number (FDR<0.1) from scRNA-seq of one-cell embryos at each log2FC (upper bound indicated for each bin).

(C) Ingenuity Pathway Analysis of down-regulated gene networks (FDR<0.10) in human one-cell embryos.

(D) Venn Diagrams showing upregulated gene overlap between previous datasets from human one-cell (1C) embryo RNA-seq and RNA-seq for two-cell (2C), four-cell (4C) and eight-cell (8C) embryos (L, Leng et al., 2019; X, Xue et al., 2013; W, Wu et al., 2018). Values in parentheses show percentages of the total in one-cell embryos. Applied parameters are provided in references or applied to datasets as follows: L-1C, L-2C, L-4C, L-8C (Leng et al., 2019); X-1C ($p_{adj}$<0.06, log2FC>2), X-2C ($p_{adj}$<0.6, log2FC>2), X-4C ($p_{adj}$<1, log2FC>2), X-8C ($p_{adj}$<0.06, log2FC>2); W-2C (log2FC>1), W-8C (log2FC>1).

(E) Venn Diagrams showing upregulated gene overlap between human one-cell embryo scRNA-seq obtained here (FDR<0.1, logFC>0.5) and data previously reported for one-cell (1C), two-cell (2C), four-cell (4C) and eight-cell (8C) embryos (L, Leng et al., 2019; X, Xue et al., 2013; W, Wu et al., 2018) or sperm intact RNA (sperm) (Sun et al., 2021) (FDR<0.1), showing (in parentheses) percentage of the total in one-cell embryos. Comparisons were of annotated genes using parameters of (D).

(F) Heatmap indicating expression trajectories of 153 of the top 200 upregulated DEGs from one-cell embryos determined by scRNA-seq in this work (FDR<0.1), relative to expression in single blastomeres from two-, four- and eight-cell embryos (Leng et al., 2019). Genes were sorted into three clusters (I, II, and III) as indicated.

See also Figure 1 and Table S1.

**Figure S2. Differential gene expression networks in human one-cell embryos**

(A) Histograms derived from scRNA-seq showing endogenous retrovirus (*hERV*) loci upregulated in human one-cell embryos. Locus coordinates are shown on the x-axis.

(B) Histograms derived from scRNA-seq showing endogenous retrovirus (*hERV*) loci down-regulated in human one-cell embryos. Locus coordinates are given on the x-axis.

(C) Pie chart showing functional classes of upregulated DEGs (FDR<0.10) related to molecular mechanisms of cancer (Ingenuity).

(D) Upstream transcription activators predicted by Ingenuity Pathway Analysis (IPA) of upregulated DEGs in human one-cell embryos (FDR<0.10).

(E-H) Upstream transcription regulators and associated networks inferred by IPA of upregulated gene networks (FDR<0.10) in human one-cell embryos for RABL6 (E), FOXM1 (F), YAP1 (G) and TP53 (H).

(I) Genes in the E2F4 cluster identified by IPA.

See also Figure 2 and Table S1.

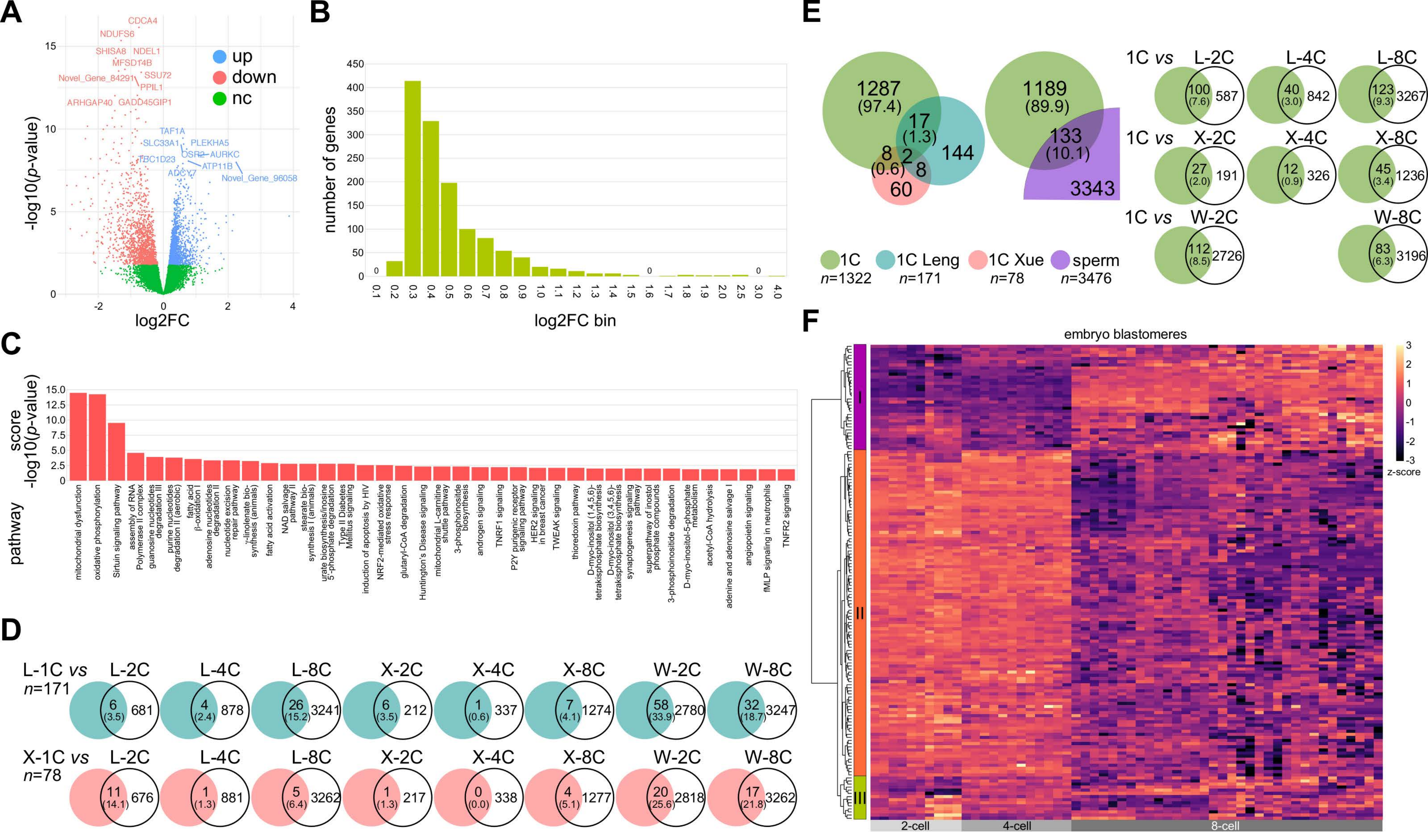


**Figure S1**
**Asami et al.**

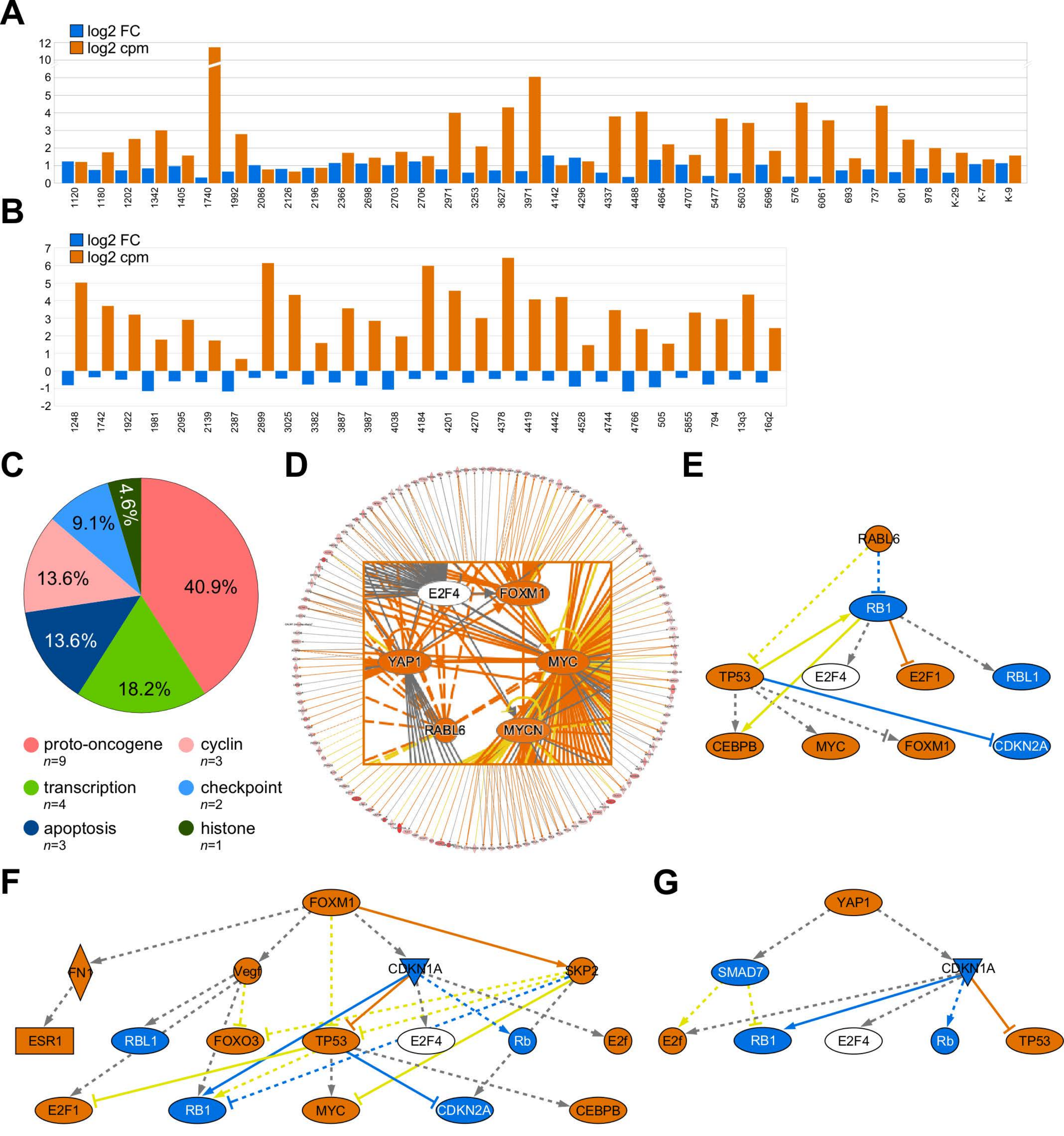


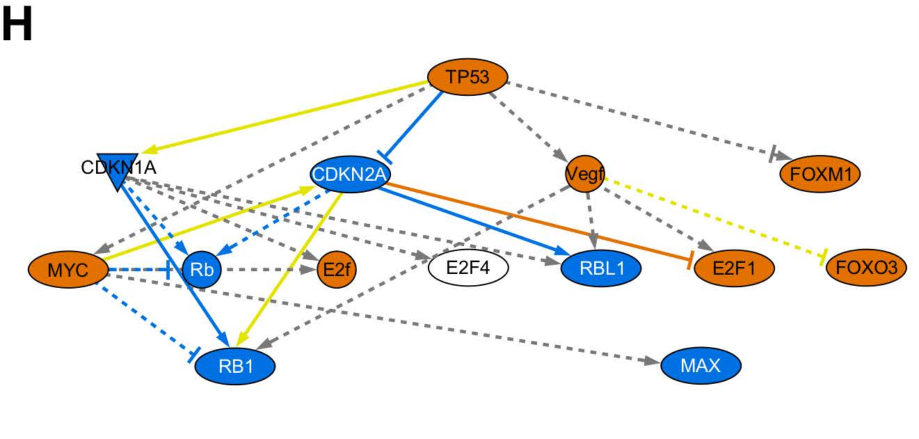


I

| E2F4 (6.48e-12) | | |
|---|---|---|
| ANLN | H2AC6 | POLA1 |
| AURKB | H2AX | PRC1 |
| BARD1 | HMGB3 | PRKDC |
| C5orf22 | HMMR | PTTG1 |
| CALM1 | KIF4A | RAD54L |
| CCDC77 | MAD2L1 | RBL2 |
| CCNE1 | MCM10 | TMPO |
| CCNE2 | MCM6 | TOP2A |
| CDK1 | NEK2 | TP53INP1 |
| DDX11 | NPM1 | TPX2 |
| DROSHA | ORC1 | TRMT13 |
| FDFT1 | PCNA | ZNF267 |

**Figure S2**
**Asami et al.**

**Table S2  PCR Primers used in this work, related to Figures 1 and 2**

| Sequence | Forward (5'→3') | Reverse (5'→3') |
|---|---|---|
| H3F3A | ACAAAAGCCGCTCGCAAGAGTG | TTTCTCGCACCAGACGCTGGAA |
| CDH15 | AGCGTATCCGAGAACCACAAGC | CTGGATGCTGTAGATGACGCTG |
| TAF1A | TCTGTGCTCAGTGGTGCAGGAA | TGCCATTGGTGCTTCAGCAGAG |
| ZCCHC8 | TTTCCAGCAGCGATACCACGCA | GGAAGACTCTTGTCTGTCACACC |
| AIF1L | CCTTCCAGAAAAGCTCACAGCC | CTTCATCTCCAGGTGGGTCTTG |
| TXNIP | CAGCAGTGCAAACAGACTTCGG | CTGAGGAAGCTCAAAGCCGAAC |
| TIGD5 | GTGCTGTTCCTGTCCAAAGGCA | AGGACACAGCCAGTCGCAGCA |
| CMTR2 | CTGACCACTCTTGGAAACGGTG | GACTTCGGAGTTTCCTGCCTTG |
| RIPK4 | CTGAAACCGAGGACCTGTGTGA | GCTGTAGTCGTTATCGAAGGTGG |
| OPRM1 | CGTCAGAACACTAGAGACCACC | CTTGGTGAAGGTCGGAATGGCA |
| RAP2C | GAGCAGTTTGCCTCCATGAGAG | CCTACTAGGATTAGTGGGACTTTT |
| PIAS3 | TGGTGATGAGTTTCCGGGT | GGAAAGCGTCGTCGGTAA |
| UGDH | TGTGATGGTGCCCATGCTGTTG | GTCCATCGAAGATAAAGGCTGGC |
| NFKBIA | TCCACTCCATCCTGAAGGCTAC | CAAGGACACCAAAAGCTCCACG |
| CCNE1 | TGTGTCCTGGATGTTGACTGCC | CTCTATGTCGCACCACTGATACC |

**Table S3  Human embryos used in this work**

| # | Patient | Sample | Analysis | Ethnicity | Age (yr) | Yield (nM) | Useable reads | Normalization factor (TMM) | Genes detected (≥1cpm) |
|---|---|---|---|---|---|---|---|---|---|
| 1 | 1 | 2PN | RNAseq | Asian | nd | 61.7 | 68635512 | 0.9692079 | 15709 |
| 2 | 1 | 2PN | RNAseq | Asian | nd | 60.8 | 65181145 | 0.9699501 | 15601 |
| 3 | 1 | 2PN | RNAseq | Asian | nd | 48.8 | 88623885 | 0.9031541 | 15732 |
| 4 | 2 | 2PN | RNAseq | Caucasian | nd | 31.2 | 46084528 | 1.001886 | 15726 |
| 5 | 2 | 2PN | RNAseq | Caucasian | nd | 3.1 | Amplification failure | nd | nd |
| 6 | 3 | 2PN | RNAseq | Caucasian | nd | 54.8 | 67065772 | 0.981549 | 15763 |
| 7 | 4 | 2PN | RNAseq | Caucasian | nd | 52.9 | 76729549 | 0.957278 | 15630 |
| 8 | 4 | 2PN | RNAseq | Caucasian | nd | 46.2 | 76189001 | 0.944969 | 15762 |
| 9 | 4 | 2PN | RNAseq | Caucasian | nd | 51.6 | 73264323 | 0.9851035 | 15918 |
| 10 | 5 | 2PN | RNAseq | Caucasian | nd | 43.7 | 75180066 | 0.9368282 | 15767 |
| 11 | 5 | 2PN | RNAseq | Caucasian | nd | 46.6 | 73222174 | 0.9957537 | 15831 |
| 12 | 6 | 2PN | RNAseq | Caucasian | nd | 67.1 | 66404656 | 0.9754091 | 15753 |
| 13 | 6 | 2PN | RNAseq | Caucasian | nd | 43.5 | 58715420 | 0.9919022 | 15602 |
| 14 | 7 | mII | RNAseq | Caucasian | nd | 46.7 | 72657324 | 0.9876494 | 15585 |
| 15 | 7 | mII | RNAseq | Caucasian | nd | 15.4 | Excluded outlier | nd | nd |
| 16 | 8 | mII | RNAseq | Caucasian | nd | 66.1 | 65324304 | 1.0415315 | 15825 |
| 17 | 8 | mII | RNAseq | Caucasian | nd | 52.2 | 72466449 | 1.0608596 | 15885 |
| 18 | 9 | mII | RNAseq | Caucasian | <30 | 72.5 | Excluded outlier | nd | nd |
| 19 | 9 | mII | RNAseq | Caucasian | <30 | 25.2 | 54705074 | 1.0171751 | 15455 |
| 20 | 10 | mII | RNAseq | Caucasian | 24.5 | 68.2 | 51126979 | 1.0306027 | 15860 |
| 21 | 10 | mII | RNAseq | Caucasian | 24.5 | 57.8 | 55244789 | 1.026353 | 15818 |
| 22 | 11 | mII | RNAseq | African American/Hispanic | 31 | 94.2 | 67438079 | 1.0240237 | 15812 |
| 23 | 11 | mII | RNAseq | African American/Hispanic | 31 | 97.4 | 61061212 | 1.0471581 | 15985 |
| 24 | 12 | mII | RNAseq | Caucasian | 22.5 | 46.3 | 69363653 | 1.0382324 | 15837 |

| | | | | | | | | | |
|---|---|---|---|---|---|---|---|---|---|
| 25 | 12 | mII | RNAseq | Caucasian | 22.5 | 70.1 | 65550194 | 1.0569761 | 15920 |
| 26 | 13 | mII | RNAseq | Caucasian | 25 | 21.1 | 67377209 | 1.0439114 | 15942 |
| 27 | 13 | mII | RNAseq | Caucasian | 25 | 35.8 | 54760799 | 1.0324617 | 15977 |
| 28 | 7 | mII | qPCR | Caucasian | nd | | | | |
| 29 | 7 | mII | qPCR | Caucasian | nd | | | | |
| 30 | 11 | mII | qPCR | African American/Hispanic | 31 | | | | |
| 31 | 8 | mII | qPCR | Caucasian | nd | | | | |
| 32 | 10 | mII | qPCR | Caucasian | 24.5 | | | | |
| 33 | 1 | 2PN | qPCR | Asian | nd | | | | |
| 34 | 1 | 2PN | qPCR | Asian | nd | | | | |
| 35 | 1 | 2PN | qPCR | Asian | nd | | | | |
| 36 | 1 | 2PN | qPCR | Asian | nd | | | | |
| 37 | 1 | 2PN | qPCR | Asian | nd | | | | |
| 38 | 5 | 2PN | qPCR | Caucasian | nd | | | | |
| 39 | 14 | 2PN | qPCR | Unknown | nd | | | | |
| 40 | 15 | 3PN | qPCR | Unknown | nd | | | | |
| 41 | 16 | 3PN | qPCR | Unknown | nd | | | | |
| 42 | 3 | 3PN | qPCR | Caucasian | nd | | | | |
| 43 | 17 | 1PN | qPCR | Unknown | nd | | | | |
| 44 | 15 | 1PN | qPCR | Unknown | nd | | | | |
| 45 | 18 | 1PN | qPCR | Unknown | nd | | | | |

Abbreviations: 1PN, 2PN, 3PN, respectively monopronuclear, bipronuclear and tripronuclear human one-cell embryos; nd, no data; TMM, trim-mean of M-values; cpm, counts per million.
