# Supplementary material for: Human embryonic genome activation initiates at the one-cell stage
Source: Cell Stem Cell. 2022 Feb 3;29(2):209–216.e4. doi: 10.1016/j.stem.2021.11.012 (PMC8826644; doi:10.1016/j.stem.2021.11.012)
Supplement: Document S2. Article plus supplemental information [file mmc3.pdf]

Cell Stem Cell

Brief Report

Human embryonic genome activation initiates at the one-cell stage

Graphical abstract

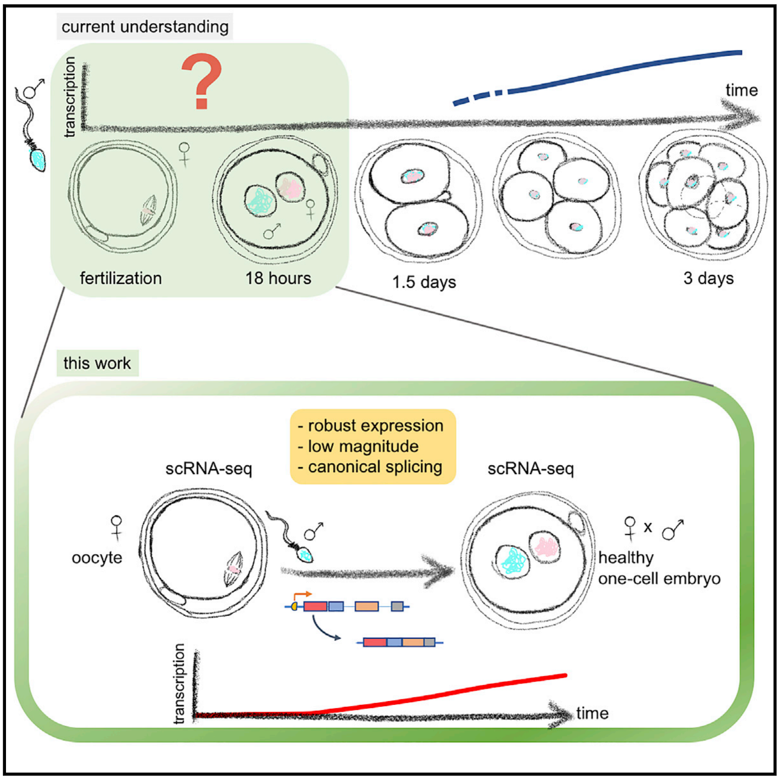

Highlights

- Gene expression initiates at the one-cell stage in human embryos
- Expression is of low magnitude but remains elevated until the eight-cell stage
- Upregulated transcripts are spliced and correspond to embryonic processes
- Upregulation is disrupted in morphologically abnormal one-cell embryos

Authors

Maki Asami, Brian Y.H. Lam,
Marcella K. Ma, ...,
Matthew D. VerMilyea, Giles S.H. Yeo,
Anthony C.F. Perry

Correspondence

mvermilyea@ovationfertility.com
(M.D.V.),
gshy2@cam.ac.uk (G.S.H.Y.),
perry135@aol.com (A.C.F.P.)

In brief

It is not known when genes are first active in newly formed human embryos. Asami and colleagues show that this activity begins soon after fertilization, at the one-cell stage. Gene activation mirrors early embryonic processes, is disrupted in abnormal embryos, and predicts links to cancer-associated transcription regulators.

Asami et al., 2022, Cell Stem Cell *29*, 209–216
February 3, 2022 © 2021 The Author(s). Published by Elsevier Inc.
https://doi.org/10.1016/j.stem.2021.11.012

Cell Stem Cell

CellPress
OPEN ACCESS

Brief Report

Human embryonic genome activation initiates at the one-cell stage

Maki Asami,[1,4] Brian Y.H. Lam,[2,4] Marcella K. Ma,[2] Kara Rainbow,[2] Stefanie Braun,[3] Matthew D. VerMilyea,[3,*] Giles S.H. Yeo,[2,*] and Anthony C.F. Perry[1,5,*]
[1]Laboratory of Mammalian Molecular Embryology, Department of Biology and Biochemistry, University of Bath, Bath BA2 7AY, England
[2]MRC Metabolic Diseases Unit, Wellcome-MRC Institute of Metabolic Science, Addenbrooke's Hospital, University of Cambridge, Cambridge CB2 0QQ, England
[3]Ovation Fertility Austin, Embryology and Andrology Laboratories, Austin, TX 78731, USA
[4]These authors contributed equally
[5]Lead contact
*Correspondence: mvermilyea@ovationfertility.com (M.D.V.), gshy2@cam.ac.uk (G.S.H.Y.), perry135@aol.com (A.C.F.P.)
https://doi.org/10.1016/j.stem.2021.11.012

SUMMARY

In human embryos, the initiation of transcription (embryonic genome activation [EGA]) occurs by the eight-cell stage, but its exact timing and profile are unclear. To address this, we profiled gene expression at depth in human metaphase II oocytes and bipronuclear (2PN) one-cell embryos. High-resolution single-cell RNA sequencing revealed previously inaccessible oocyte-to-embryo gene expression changes. This confirmed transcript depletion following fertilization (maternal RNA degradation) but also uncovered low-magnitude upregulation of hundreds of spliced transcripts. Gene expression analysis predicted embryonic processes including cell-cycle progression and chromosome maintenance as well as transcriptional activators that included cancer-associated gene regulators. Transcription was disrupted in abnormal monopronuclear (1PN) and tripronuclear (3PN) one-cell embryos. These findings indicate that human embryonic transcription initiates at the one-cell stage, sooner than previously thought. The pattern of gene upregulation promises to illuminate processes involved at the onset of human development, with implications for epigenetic inheritance, stem-cell-derived embryos, and cancer.

INTRODUCTION

Fertilizing spermatozoa and metaphase II (mII) oocytes are transcriptionally quiescent (Zhou and Dean, 2015). The first transcription in newly formed embryos is known as embryonic genome activation (EGA), but its onset, timing, and profile are poorly understood (Jukam et al., 2017). In human embryos, EGA is held to have occurred by the eight-cell stage, up to ~68 h (~3 days) after fertilization (Braude et al., 1988; Leng et al., 2019; Tesarík et al., 1988; Vassena et al., 2011; Xue et al., 2013; Yan et al., 2013), but this model is likely incomplete. First, it does not accommodate hints that transcription initiates earlier (Leng et al., 2019; Wu et al., 2018; Xue et al., 2013; Yan et al., 2013), albeit previous analyses are restricted by poor signal-to-noise ratios, low embryo or donor numbers, and RNA-sequencing (RNA-seq) library preparation protocols that reflect mRNA polyadenylation status (recruitment) (Blower et al., 2013; Oh et al., 2000; Temeles and Schultz, 1997). Second, the model does not explain how the embryo genome is maintained in a transcriptionally silent state during cell proliferation to the eight-cell stage (Alpha Scientists in Reproductive Medicine and ESHRE Special Interest Group of Embryology, 2011). Third, no mechanism has been proposed that explains how maternal factor activity required for early development is regulated at different phases of the first two-to-three cell cycles in the absence of endogenous transcription. Fourth, the model does not address the cue that instigates transcription, which can be provided either *in vivo* or *in vitro* or whether it is the cumulative consequence of transcription-independent processes. We, therefore, evaluated the open possibility that gene expression is triggered after fertilization in human one-cell embryos.

RESULTS

We sought to determine the gene expression profile of human one-cell embryos and relate it to development (Figure 1A). Human mII oocytes (n = 12, from seven donors, aged 22.5–31) and bipronuclear (2PN) one-cell embryos (n = 12, from six couples, with no oocyte donor overlap) from various ethnic backgrounds and that appeared healthy (Figure 1B) were subjected to single-cell RNA-seq (scRNA-seq) that avoided poly(A) capture and its attendant potential library bias (Blower et al., 2013; Oh et al., 2000; Temeles and Schultz, 1997). Whole-transcriptome amplification produced indistinguishable yields between oocytes and one-cell embryos (p = 0.595), and in-depth scRNA-seq yielded a mean of 66.3 million reads per cell. Principal component analysis, coupled with t-distributed

© 2021 The Author(s). Published by Elsevier Inc.
This is an open access article under the CC BY license (http://creativecommons.org/licenses/by/4.0/).

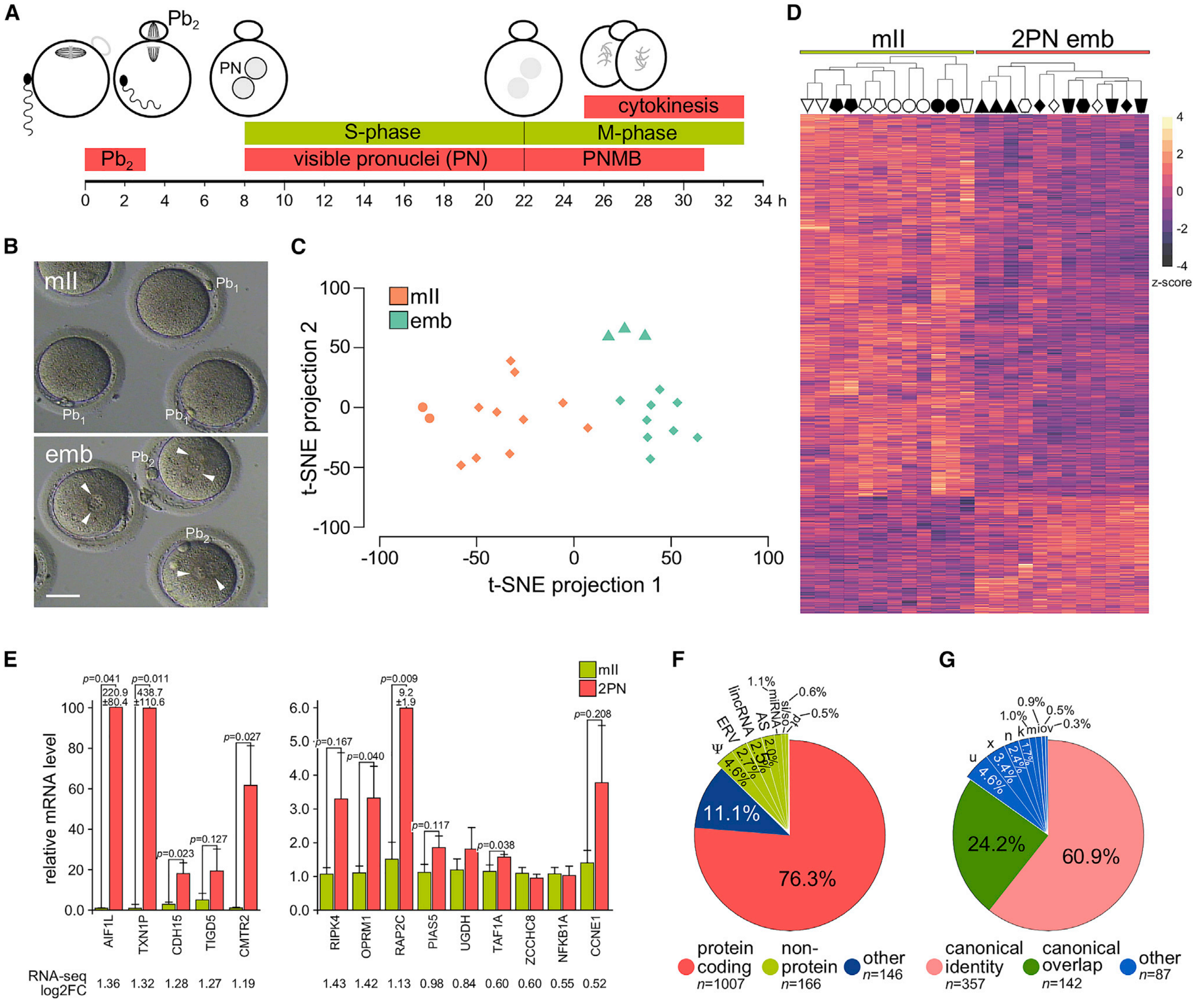

Figure 1. Human embryonic transcription initiates at the one-cell stage

(A) Schematic of human one-cell embryo development at the times after fertilization (Capmany et al., 1996). Pb_1, first polar body; Pb_2, second polar body; PN, pronuclei; PNMB, pronuclear membrane breakdown.

(B) Brightfield images of representative human metaphase II oocytes (mII) and bipronuclear one-cell embryos (emb). Arrowheads indicate pronuclei. Pb_1, first polar body; Pb_2, second polar body. Scale bar, 50 μm.

(C) t-SNE analysis (without filtering) of single-cell RNA-seq data for human mII oocytes (mII, n = 12) and bipronuclear (2PN) one-cell embryos (emb, n = 12). Circles correspond to oocytes from an African American/Hispanic donor and triangles correspond to embryos from Asian donors, with all other oocytes and embryos being of Caucasian origin and filled triangles and circles corresponding to symbols of (D).

(D) Heatmap showing changes in gene expression levels (FDR < 0.1, $log_2FC > 0.58$) in human mII oocytes (mII) (n = 12) and bipronuclear (2PN) one-cell embryos (emb; n = 12) of (C), indicating donors (top) and the Z score scale (−4 to 4). Each patient is represented by a symbol to indicate the provenance of oocytes and embryos.

(E) Single-cell qPCR of upregulated DEG transcripts in individual human oocytes (mII; n $\geq$ 3 independent biological replicates) and 2PN one-cell embryos (emb; n $\geq$ 3 independent biological replicates) (FDR < 0.1, $log_2FC > 0.5$). Different oocytes and embryos were used to those of (D). Corresponding log_2FC values from RNA-seq are indicated beneath histograms. Primer pairs flanked exon junctions except in the cases of *TIGD5* and *NFKB1A* (which have a single exon) and *PIAS3*. Values are ± SEM and normalized against mII oocytes.

(F) Pie chart showing functional classes of upregulated DEGs (FDR < 0.1, cpm > 1.0). Ψ, pseudogene; ERV, endogenous retrovirus; lncRNA, long non-coding RNA; AS, antisense; miRNA, microRNA; si/so, sense-intronic/sense-overlapping; pt, processed transcript.

(G) Pie chart showing processing classes (FDR < 0.34) of upregulated DEGs (p < 0.05; cpm > 1.0). Letters indicate Ingenuity codes.

See also Figure S1 and Table S1.

stochastic neighbor embedding (t-SNE), segregated transcriptomes into discrete oocyte and embryo groups (Figure 1C), and comparison revealed 2,879 differentially expressed genes (DEGs; FDR < 0.1; Figures 1D and S1A). The number of DEGs inversely correlated with fold-change (FC, log_2FC; Figure S1B), and there were 1,395 DEGs with an absolute log_2FC

CellPress
OPEN ACCESS

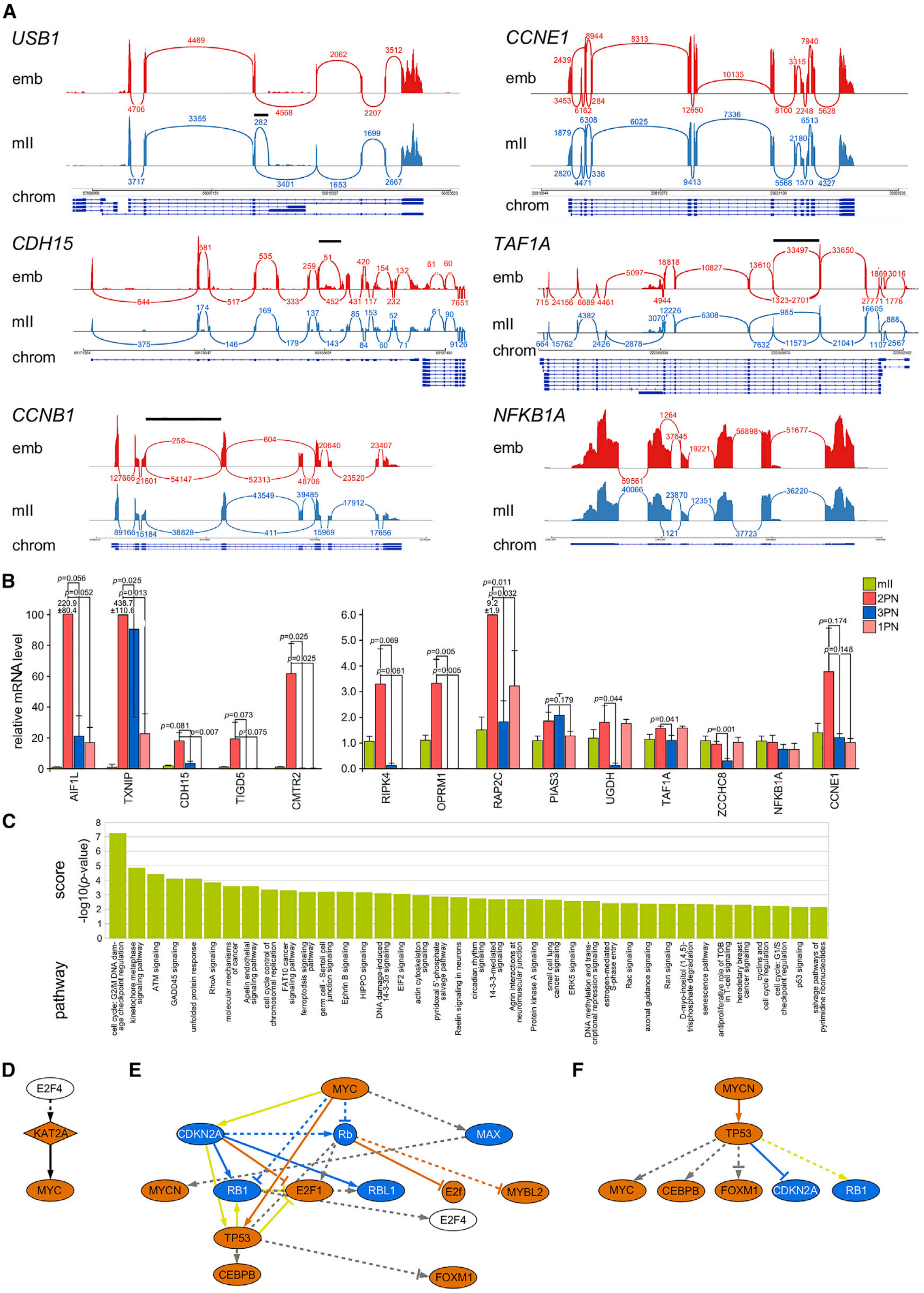

Figure 2. Human one-cell upregulated gene characteristics and pathways

(A) Raw scRNA-seq density plots (Sashimi plots) along exons and exon junctions. Arcs representing splice junctions connecting exons and display the number of reads split across the junction (junction depth) in mII oocytes (mII) and 2PN one-cell embryos (emb). Genomic coordinates (chrom) and gene annotation tracks are aligned beneath each respective plot. Solid black bars above plots indicate regions of potential alternative splicing.

(legend continued on next page)

of ≥ 0.5 (of which 1,081 survived an FDR cutoff of 0.05). Expression levels of 1,557 genes (54.1% of DEGs; FDR < 0.1) decreased in one-cell embryos, reflecting maternal transcript degradation (Figures 1D and S1C) (Alizadeh et al., 2005). These excluded orthologs of classically down-regulated mouse transcripts, including *MOS* and *GDF9*, consistent with relatively slow human maternal transcript degradation lasting several cell cycles (i.e., days) (Leng et al., 2019; Paynton et al., 1988; Petropoulos et al., 2016; Sha et al., 2020; Xue et al., 2013). Outputs from Ingenuity pathway analysis (IPA) (Krämer et al., 2014) corresponded to the initiation of embryogenesis and conclusion of gametogenesis (Figure 1A; Table S1).

In addition to down-regulation, expression of 1,322 genes increased in one-cell embryos compared with mII oocytes (FDR < 0.1) (Figure 1D). To test this, we assessed the overlap between upregulated DEGs and 657 transcripts common to four datasets of stably expressed human genes (n = 6,040 ± 2,549 genes; Eisenberg and Levanon, 2013; Lin et al., 2019; Petropoulos et al., 2016). Of these, 542 were expressed in one-cell embryos, most (90.4%) at levels indistinguishable from those in oocytes. Single-cell qPCR of upregulated DEGs in different one-cell embryos mirrored increases revealed by scRNA-seq ($log_2FC > 0.5$; $n \geq 3$ different embryos per target; n = 14 targets; Figure 1E). Most (76.3%) upregulated DEGs encoded annotated proteins (Figure 1F) and transcriptome re-assembly *de novo* predicted the use of canonical transcription start sites in 60.9% (n = 357) of cases and normative splicing in 85.1% (Figure 1G). Sashimi plots demonstrated increases in the levels of mature mRNA transcripts spliced at canonical exon junctions in one-cell embryos, with evidence of embryo-specific alternative splicing (Figure 2A). Exon-flanking qPCR further corroborated mature, spliced mRNA level increases (Figure 1E). These findings reveal the onset of human EGA in one-cell embryos to produce canonically spliced mRNA transcripts.

The degree of overlap between upregulated DEGs and previous expression data for cleavage-stage human embryos (Figures S1D and S1E) possibly reflected protocol differences (e.g., poly(A) capture in library preparation), donor ethnicity, and timing of gamete and embryo collection (Leng et al., 2019; Xue et al., 2013). Only 3.8% of 3,476 human-sperm-intact RNAs (Sun et al., 2021) corresponded to upregulated DEGs (FDR < 0.1; Figure S1E), although sperm-associated RNA may not enter mII oocytes (Amanai et al., 2006; Asami et al., 2020; Zhou et al., 2019). Of the top 200 upregulated genes (FDR < 0.1), 153 were differentially expressed in two-, four-, and eight-cell embryos (Leng et al., 2019) (Figure S1F). Most (119/153; 77.8%) exhibited sustained expression throughout two-to-four-cell stages and markedly declined by the eight-cell stage (clusters II and III, Figure S1F).

We investigated expression of upregulated transcripts in tripronuclear (3PN) one-cell embryos, which have been adopted for genome editing studies (Kang et al., 2016; Li et al., 2017; Liang et al., 2015). Of 14 targets upregulated in 2PN embryos that we evaluated, levels of 12 (85.7%) were lower (p < 0.2) in 3PN embryos (Figure 2B). This suggests that EGA initiation was disrupted in most 3PN one-cell embryos, which undergo developmental failure (Kola et al., 1987; Mutia et al., 2019). Moreover, expression of 10 of the 14 targets (71.4%; p < 0.2) was lower in monopronuclear (1PN) one-cell embryos (Figure 2B).

IPA of upregulated DEGs indicated that expressed gene function corresponded to developmental processes in healthy 2PN embryos (Figures 1A and 2C; Table S1). These included ATM activation, which induces G2-/M-phase arrest in response to DNA damage in the mouse (Wang et al., 2013), protective redox-dependent systems (Perreault et al., 1988; Yanagimachi, 1994), and chromosome segregation, consistent with the elimination of aneuploid two-cell embryos (Pauerova et al., 2020; Schatten et al., 1988). Upregulated DEGs associated with early developmental arrest included *WEE2* (p = 0.006; Sang et al., 2018) and *BTG4* (p = 0.015; Zheng et al., 2020) as well as disease-associated nuclear genes involved in mitochondrial function (e.g., *TFB2M*, p = 6.71e-05; *MFN1*, p = 1.34e-05), consistent with important early embryonic roles played by mitochondria (Facucho-Oliveira and St John, 2009; McConnell and Petrie, 2004; Rusecka et al., 2018).

We found no evidence for major upregulation of LINE-1 retrotransposons (Percharde et al., 2018) or transcription factors (TFs) thought to drive cleavage-stage EGA, including *OCT4* (Gao et al., 2018), *LEUTX* (Jouhilahti et al., 2016), and *DUX4* (De Iaco et al., 2017; Hendrickson et al., 2017). However, upregulated DEGs included 63 human endogenous retrovirus (*hERV*) loci (Figures S2A and S2B). Trans-activators of EGA initiation predicted by IPA (FDR < 0.1) included MYC (p = 1.94e-6), MYCN (p = 4.54e-8), RABL6 (p = 0.02), FYN (p = 0.05), and E2F4 (p = 6.48e-12) (Figures 2D–2F and S2C–S2I). Transcripts for a third of these (33/97) were detected by scRNA-seq in mII oocytes and down-regulation of E2F4 correlates with one-cell arrest (Suo et al., 2018).

DISCUSSION

These findings have several implications. First, they suggest that transcription is instated soon after fertilization, during meiotic progression and gamete reprogramming, which should illuminate mechanisms that coordinate chromatin remodeling, the cell cycle, and transcription complex assembly. Second, they provide a readout of epigenetic states that support embryonic transcription and locate genomic addresses of active chromatin. Third, they indicate that decoding gamete

(B) qPCR for transcripts in individual human monopronuclear (1PN) and tripronuclear (3PN) one-cell embryos ($3 \geq n \geq 6$ biologically independent oocytes or embryos per target). Values for metaphase II oocytes (mII) and bipronuclear one-cell embryos (2PN) from Figure 1E are included for comparison. Values are ± SEM and normalized against mII oocytes (~1.0). Unpaired t tests indicate p < 0.2.

(C) Ingenuity pathway analysis (IPA) of gene networks upregulated (FDR < 0.1, $log_2FC > 0$) in 2PN one-cell embryos.

(D–F) Upstream transcription regulators inferred by IPA of upregulated gene networks (FDR < 0.1, $log_2FC > 0$) in 2PN one-cell embryos for E2F4 (D), MYC (E), and MYCN (F).

See also Figure S2 and Table S1.

chromatin modifications acutely after fertilization might mediate epigenetic inheritance. Fourth, commencement of human gene expression in one-cell embryos accommodates previous hints of early transcription (Wu et al., 2018; Yan et al., 2013; Xue et al., 2013), circumvents protracted genome latency and autonomous regulation, and suggests the trigger for EGA: fertilization.

Other contexts relevant to cellular potency also manifest small magnitude transcript-level changes that may be common in cellular potency transitions. Trans-activation by MYC induces relevant target gene expression less than two-fold (Baluapuri et al., 2020; Nilsson et al., 2005) and mouse pluripotency factor; Esrrb apparently causes less than two-fold upregulation of embryonic-stem-cell-specific genes (Chronis et al., 2017). The average negative effect of the transcription regulator, Polycomb, is about two-fold (Berrozpe et al., 2017), and its associated histone modification, H3K27me3, modulates cellular potency and imprinting (Bernstein et al., 2006; Santini et al., 2021). Several lines of evidence suggest that transcription at the one-cell stage is functional. Upregulated transcripts exhibited a stereotypical pattern that corresponded to early embryonic processes (Perry and Verlhac, 2008; Yanagimachi, 1994; Zhou and Dean, 2015), encoded protein, and utilized canonical exons and transcriptional start sites. Predicted transcription regulator functions mapped to one-cell embryonic processes and the regulation of cancer (Figures 1A, 2C–2F, and S2C–S2I). The switch at fertilization that activates presumptive maternal TFs may involve phospho-relay signaling (Perry and Verlhac, 2008), as MYC, MYCN, FOXM1, E2F4, and others are regulated by kinases (Joshi et al., 2013; Morillo et al., 2012; Sjostrom et al., 2005; Vervoorts et al., 2006). Trajectory analysis (Figure S1F) implied that TFs responsible for early expression are deactivated during the four-to-eight-cell transition, coinciding with a major wave of EGA.

Transcriptional initiation in human one-cell embryos also has clinical implications. Disruption of cancer-associated genes might manifest as both impaired fertility and cancer, and female infertility is indeed associated with ~15% elevated cancer risk (Murugappan et al., 2019). It is also possible that parentally inherited epigenetic marks (e.g., those associated with acquired obesogenic traits) affect gene expression immediately after fertilization (Huypens et al., 2016; Samata et al., 2020). Maternal factors required to initiate EGA may include polar body markers of oocyte quality (Klatsky et al., 2010; Reich et al., 2011; Swain and Pool, 2008; VerMilyea et al., 2011) and reveal processes in artificial oocytes or embryos necessary for embryonic transcription and totipotency (Yamashiro et al., 2018). Although stem-cell-derived human embryos (blastoids) may one day bypass embryonic totipotency (Liu et al., 2021; Yanagida et al., 2021; Yu et al., 2021), EGA initiation may alternatively be indispensable for normative development. Finally, evaluating genome editing in 3PN embryos (Kang et al., 2016; Liang et al., 2015; Li et al., 2017) should accommodate disrupted gene expression to have clinical utility (Perry, 2000; Yeh et al., 2019).

Limitations of the study

Human 2PN one-cell embryos that may legitimately be used for research are extremely rare, in part reflecting current standard practice among assisted reproduction facilities. This study sourced archival 2PN embryos, placing constraints on sample availability that, *inter alia*, precluded corroborative analysis orthogonal to high-resolution scRNA-seq, including inhibitor studies (e.g., to block transcription by RNA polymerase II) and confirmatory immunofluorescence (e.g., of predicted maternal TFs). The findings are likely to be evaluated in more tractable (e.g., mouse) models in the foreseeable future, and perhaps eventually in closely related primate (e.g., baboon) models.

STAR★METHODS

Detailed methods are provided in the online version of this paper and include the following:

- KEY RESOURCES TABLE
- RESOURCE AVAILABILITY
  - Lead contact
  - Materials availability
  - Data and code availability
- EXPERIMENTAL MODEL AND SUBJECT DETAILS
  - Human oocyte and embryo sample collection
- METHOD DETAILS
  - Human metaphase II oocytes and one-cell embryos (zygotes)
  - Single-cell RNA sequencing (scRNA-seq) of oocytes and one-cell embryos
  - Ratiometric real-time-PCR (qPCR)
- QUANTIFICATION AND STATISTICAL ANALYSIS
  - Bioinformatic analysis
  - Statistical analysis

SUPPLEMENTAL INFORMATION

Supplemental information can be found online at https://doi.org/10.1016/j.stem.2021.11.012.

ACKNOWLEDGMENTS

We are greatly indebted to Drs. C. Klein, A. Greenfield, and C. Tickle for comments during manuscript preparation and thank J. Whitney for embryo curation. We acknowledge support to A.C.F.P. from the Biotechnology and Biological Sciences Research Council (BBSRC; BB/P009506/1) and Medical Research Council (MRC), UK (MR/N000080/1 and MR/N020294/1). B.Y.H.L. is supported by BBSRC Project Grant BB/S017593/1 and G.S.H.Y. by the MRC Metabolic Diseases Unit (MC_UU_00014/1). Next-generation sequencing was performed by the IMS Genomics and transcriptomics core facility and supported by the MRC (MC_UU_00014/5), the Wellcome Trust (208363/Z/17/Z), and the Cancer Research UK Cambridge Institute Genomics Core.

AUTHOR CONTRIBUTIONS

A.C.F.P. conceived the core idea. Embryo preparation was by M.D.V. and S.B. Single-cell whole-transcriptome amplification was performed by B.Y.H.L., K.R., and M.K.M. B.Y.H.L. designed scRNA-seq analyses, performed and evaluated by B.Y.H.L., M.K.M., and G.S.H.Y. M.A. performed qPCR. Data analysis was by M.A., B.Y.H.L., G.S.H.Y., and A.C.F.P. A.C.F.P. wrote the manuscript with input from B.Y.H.L. and M.A.

DECLARATION OF INTERESTS

The authors declare no competing interests.

Received: April 8, 2021
Revised: August 24, 2021
Accepted: November 29, 2021
Published: December 21, 2021

REFERENCES

Alizadeh, Z., Kageyama, S.-I., and Aoki, F. (2005). Degradation of maternal mRNA in mouse embryos: selective degradation of specific mRNAs after fertilization. Mol. Reprod. Dev. *72*, 281–290.

Alpha Scientists in Reproductive Medicine and ESHRE Special Interest Group of Embryology (2011). The Istanbul consensus workshop on embryo assessment: proceedings of an expert meeting. Hum. Reprod. *26*, 1270–1283.

Amanai, M., Brahmajosyula, M., and Perry, A.C.F. (2006). A restricted role for sperm-borne microRNAs in mammalian fertilization. Biol. Reprod. *75*, 877–884.

Anders, S., Pyl, P.T., and Huber, W. (2015). HTSeq–a Python framework to work with high-throughput sequencing data. Bioinformatics *31*, 166–169.

Asami, M., Lam, B.Y.H., Hoffmann, M., Suzuki, T., Lu, X., VerMilyea, M.D., Yoshida, N., Ma, M.K., Rainbow, K., Braun, S., et al. (2020). Mouse fertilization triggers a conserved transcription program in one-cell embryos. bioRxiv. https://doi.org/10.1101/2020.09.15.298018.

Baluapuri, A., Wolf, E., and Eilers, M. (2020). Target gene-independent functions of MYC oncoproteins. Nat. Rev. Mol. Cell Biol. *21*, 255–267.

Bernstein, B.E., Mikkelsen, T.S., Xie, X., Kamal, M., Huebert, D.J., Cuff, J., Fry, B., Meissner, A., Wernig, M., Plath, K., et al. (2006). A bivalent chromatin structure marks key developmental genes in embryonic stem cells. Cell *125*, 315–326.

Berrozpe, G., Bryant, G.O., Warpinski, K., Spagna, D., Narayan, S., Shah, S., and Ptashne, M. (2017). Polycomb responds to low levels of transcription. Cell Rep *20*, 785–793.

Blower, M.D., Jambhekar, A., Schwarz, D.S., and Toombs, J.A. (2013). Combining different mRNA capture methods to analyze the transcriptome: analysis of the *Xenopus laevis* transcriptome. PLoS One *8*, e77700.

Braude, P., Bolton, V., and Moore, S. (1988). Human gene expression first occurs between the four- and eight-cell stages of preimplantation development. Nature *332*, 459–461.

Camus, M., Van den Abbeel, E., Van Waesberghe, L., Wisanto, A., Devroey, P., and Van Steirteghem, A.C. (1989). Human embryo viability after freezing with dimethylsulfoxide as a cryoprotectant. Fertil. Steril. *51*, 460–465.

Capmany, G., Taylor, A., Braude, P.R., and Bolton, V.N. (1996). The timing of pronuclear formation, DNA synthesis and cleavage in the human 1-cell embryo. Mol. Hum. Reprod. *2*, 299–306.

Chronis, C., Fiziev, P., Papp, B., Butz, S., Bonora, G., Sabri, S., Ernst, J., and Plath, K. (2017). Cooperative binding of transcription factors orchestrates reprogramming. Cell *168*, 442–459.e20.

De Iaco, A., Planet, E., Coluccio, A., Verp, S., Duc, J., and Trono, D. (2017). DUX-family transcription factors regulate zygotic genome activation in placental mammals. Nat. Genet. *49*, 941–945.

Dobin, A., Davis, C.A., Schlesinger, F., Drenkow, J., Zaleski, C., Jha, S., Batut, P., Chaisson, M., and Gingeras, T.R. (2013). STAR: ultrafast universal RNA-seq aligner. Bioinformatics *29*, 15–21.

Eisenberg, E., and Levanon, E.Y. (2013). Human housekeeping genes, revisited. Trends Genet *29*, 569–574.

Evans, C., Hardin, J., and Stoebel, D.M. (2018). Selecting between-sample RNA-Seq normalization methods from the perspective of their assumptions. Brief. Bioinform. *19*, 776–792.

Facucho-Oliveira, J.M., and St John, J.C. (2009). The relationship between pluripotency and mitochondrial DNA proliferation during early embryo development and embryonic stem cell differentiation. Stem Cell Rev. Rep. *5*, 140–158.

Gao, L., Wu, K., Liu, Z., Yao, X., Yuan, S., Tao, W., Yi, L., Yu, G., Hou, Z., Fan, D., et al. (2018). Chromatin accessibility landscape in human early embryos and its association with evolution. Cell *173*, 248–259.e15.

Gook, D.A., Osborn, S.M., and Johnston, W.I. (1993). Cryopreservation of mouse and human oocytes using 1,2-propanediol and the configuration of the meiotic spindle. Hum. Reprod. *8*, 1101–1109.

Hendrickson, P.G., Doráis, J.A., Grow, E.J., Whiddon, J.L., Lim, J.W., Wike, C.L., Weaver, B.D., Pflueger, C., Emery, B.R., Wilcox, A.L., et al. (2017). Conserved roles of mouse Dux and human Dux4 in activating cleavage-stage genes and MERVL/HERVL retrotransposons. Nat. Genet. *49*, 925–934.

Huypens, P., Sass, S., Wu, M., Dyckhoff, D., Tschöp, M., Theis, F., Marschall, S., Hrabě de Angelis, M., and Beckers, J. (2016). Epigenetic germline inheritance of diet-induced obesity and insulin resistance. Nat. Genet. *48*, 497–499.

Joshi, K., Banasavadi-Siddegowda, Y., Mo, X., Kim, S.H., Mao, P., Kig, C., Nardini, D., Sobol, R.W., Chow, L.M., Kornblum, H.I., et al. (2013). MELK-dependent FOXM1 phosphorylation is essential for proliferation of glioma stem cells. Stem Cells *31*, 1051–1063.

Jouhilahti, E.M., Madissoon, E., Vesterlund, L., Töhönen, V., Krjutškov, K., Plaza Reyes, A., Petropoulos, S., Månsson, R., Linnarsson, S., Bürglin, T., et al. (2016). The human PRD-like homeobox gene *LEUTX* has a central role in embryo genome activation. Development *143*, 3459–3469.

Jukam, D., Shariati, S.A.M., and Skotheim, J.M. (2017). Zygotic genome activation in vertebrates. Dev. Cell *42*, 316–332.

Kang, X., He, W., Huang, Y., Yu, Q., Chen, Y., Gao, X., Sun, X., and Fan, Y. (2016). Introducing precise genetic modifications into human 3PN embryos by CRISPR/Cas-mediated genome editing. J. Assist. Reprod. Genet. *33*, 581–588.

Katz, Y., Wang, E.T., Airoldi, E.M., and Burge, C.B. (2010). Analysis and design of RNA sequencing experiments for identifying isoform regulation. Nat. Methods *7*, 1009–1015.

Klatsky, P.C., Wessel, G.M., and Carson, S.A. (2010). Detection and quantification of mRNA in single human polar bodies: a minimally invasive test of gene expression during oogenesis. Mol. Hum. Reprod. *16*, 938–943.

Kola, I., Trounson, A., Dawson, G., and Rogers, P. (1987). Tripronuclear human oocytes: altered cleavage patterns and subsequent karyotypic analysis of embryos. Biol. Reprod. *37*, 395–401.

Krämer, A., Green, J., Pollard, J., Jr., and Tugendreich, S. (2014). Causal analysis approaches in Ingenuity Pathway Analysis. Bioinformatics *30*, 523–530.

Law, C.W., Chen, Y., Shi, W., and Smyth, G.K. (2014). voom: precision weights unlock linear model analysis tools for RNA-seq read counts. Genome Biol *15*, R29.

Leng, L., Sun, J., Huang, J., Gong, F., Yang, L., Zhang, S., Yuan, X., Fang, F., Xu, X., Luo, Y., et al. (2019). Single-cell transcriptome analysis of uniparental embryos reveals parent-of-origin effects on human preimplantation development. Cell Stem Cell *25*, 697–712.e6.

Li, G., Liu, Y., Zeng, Y., Li, J., Wang, L., Yang, G., Chen, D., Shang, X., Chen, J., Huang, X., and Liu, J. (2017). Highly efficient and precise base editing in discarded human tripronuclear embryos. Protein Cell *8*, 776–779.

Liang, P., Xu, Y., Zhang, X., Ding, C., Huang, R., Zhang, Z., Lv, J., Xie, X., Chen, Y., Li, Y., et al. (2015). CRISPR/Cas9-mediated gene editing in human tripronuclear zygotes. Protein Cell *6*, 363–372.

Lin, Y., Ghazanfar, S., Strbenac, D., Wang, A., Patrick, E., Lin, D.M., Speed, T., Yang, J.Y.H., and Yang, P. (2019). Evaluating stably expressed genes in single cells. GigaScience *8*, giz106.

Liu, X., Tan, J.P., Schröder, J., Aberkane, A., Ouyang, J.F., Mohenska, M., Lim, S.M., Sun, Y.B.Y., Chen, J., Sun, G., et al. (2021). Modelling human blastocysts by reprogramming fibroblasts into iBlastoids. Nature *591*, 627–632.

McConnell, J.M., and Petrie, L. (2004). Mitochondrial DNA turnover occurs during preimplantation development and can be modulated by environmental factors. Reprod. Biomed. Online *9*, 418–424.

Morillo, S.M., Abanto, E.P., Román, M.J., and Frade, J.M. (2012). Nerve growth factor-induced cell cycle reentry in newborn neurons is triggered by $p38^{MAPK}$-dependent E2F4 phosphorylation. Mol. Cell. Biol. *32*, 2722–2737.

Murugappan, G., Li, S., Lathi, R.B., Baker, V.L., and Eisenberg, M.L. (2019). Risk of cancer in infertile women: analysis of US claims data. Hum. Reprod. *34*, 894–902.

Mutia, K., Wiweko, B., Iffanolida, P.A., Febri, R.R., Muna, N., Riayati, O., Jasirwan, S.O., Yuningsih, T., Mansyur, E., and Hestiantoro, A. (2019). The frequency of chromosomal euploidy Among 3PN embryos. J. Reprod. Infertil. *20*, 127–131.

Nilsson, J.A., Keller, U.B., Baudino, T.A., Yang, C., Norton, S., Old, J.A., Nilsson, L.M., Neale, G., Kramer, D.L., Porter, C.W., and Cleveland, J.L. (2005). Targeting ornithine decarboxylase in Myc-induced lymphomagenesis prevents tumor formation. Cancer Cell *7*, 433–444.

Oh, B., Hwang, S., McLaughlin, J., Solter, D., and Knowles, B.B. (2000). Timely translation during the mouse oocyte-to-embryo transition. Development *127*, 3795–3803.

Pauerova, T., Radonova, L., Kovacovicova, K., Novakova, L., Skultety, M., and Anger, M. (2020). Aneuploidy during the onset of mouse embryo development. Reproduction *160*, 773–782.

Paynton, B.V., Rempel, R., and Bachvarova, R. (1988). Changes in state of adenylation and time course of degradation of maternal mRNAs during oocyte maturation and early embryonic development in the mouse. Dev. Biol. *129*, 304–314.

Percharde, M., Lin, C.J., Yin, Y., Guan, J., Peixoto, G.A., Bulut-Karslioglu, A., Biechele, S., Huang, B., Shen, X., and Ramalho-Santos, M. (2018). A LINE1-nucleolin partnership regulates early development and ESC identity. Cell *174*, 391–405.e19.

Perreault, S.D., Barbee, R.R., and Slott, V.L. (1988). Importance of glutathione in the acquisition and maintenance of sperm nuclear decondensing activity in maturing hamster oocytes. Dev. Biol. *125*, 181–186.

Perry, A.C.F. (2000). Hijacking oocyte DNA repair machinery in transgenesis? Mol. Reprod. Dev. *56*, 319–324.

Perry, A.C.F., and Verlhac, M.-H. (2008). Second meiotic arrest and exit in frogs and mice. EMBO Rep *9*, 246–251.

Pertea, M., Pertea, G.M., Antonescu, C.M., Chang, T.-C., Mendell, J.T., and Salzberg, S.L. (2015). StringTie enables improved reconstruction of a transcriptome from RNA-seq reads. Nat. Biotechnol. *33*, 290–295.

Petropoulos, S., Edsgärd, D., Reinius, B., Deng, Q., Panula, S.P., Codeluppi, S., Plaza Reyes, A., Linnarsson, S., Sandberg, R., and Lanner, F. (2016). Single-cell RNA-seq reveals lineage and X chromosome dynamics in human preimplantation embryos. Cell *165*, 1012–1026.

Reich, A., Klatsky, P., Carson, S., and Wessel, G. (2011). The transcriptome of a human polar body accurately reflects its sibling oocyte. J. Biol. Chem. *286*, 40743–40749.

Ritchie, M.E., Phipson, B., Wu, D., Hu, Y., Law, C.W., Shi, W., and Smyth, G.K. (2015). limma powers differential expression analyses for RNA-sequencing and microarray studies. Nucleic Acids Res *43*, e47.

Robinson, J.T., Thorvaldsdóttir, H., Winckler, W., Guttman, M., Lander, E.S., Getz, G., and Mesirov, J.P. (2011). Integrative genomics viewer. Nat. Biotechnol. *29*, 24–26.

Robinson, M.D., McCarthy, D.J., and Smyth, G.K. (2010). edgeR: a bioconductor package for differential expression analysis of digital gene expression data. Bioinformatics *26*, 139–140.

Robinson, M.D., and Oshlack, A. (2010). A scaling normalization method for differential expression analysis of RNA-seq data. Genome Biol *11*, R25.

Rusecka, J., Kaliszewska, M., Bartnik, E., and Tońska, K. (2018). Nuclear genes involved in mitochondrial diseases caused by instability of mitochondrial DNA. J. Appl. Genet. *59*, 43–57.

Samata, M., Alexiadis, A., Richard, G., Georgiev, P., Nuebler, J., Kulkarni, T., Renschler, G., Basilicata, M.F., Zenk, F.L., Shvedunova, M., et al. (2020). Intergenerationally maintained histone H4 lysine 16 acetylation is instructive for future gene activation. Cell *182*, 127–144.e23.

Sang, Q., Li, B., Kuang, Y., Wang, X., Zhang, Z., Chen, B., Wu, L., Lyu, Q., Fu, Y., Yan, Z., et al. (2018). Homozygous mutations in *WEE2* cause fertilization failure and female infertility. Am. J. Hum. Genet. *102*, 649–657.

Santini, L., Halbritter, F., Titz-Teixeira, F., Suzuki, T., Asami, M., Ma, X., Ramesmayer, J., Lackner, A., Warr, N., Pauler, F., et al. (2021). Genomic imprinting in mouse blastocysts is predominantly associated with H3K27me3. Nat. Commun. *12*, 3804.

Schatten, G., Simerly, C., Palmer, D.K., Margolis, R.L., Maul, G., Andrews, B.S., and Schatten, H. (1988). Kinetochore appearance during meiosis, fertilization and mitosis in mouse oocytes and zygotes. Chromosoma *96*, 341–352.

Sha, Q.-Q., Zheng, W., Wu, Y.-W., Li, S., Guo, L., Zhang, S., Lin, G., Ou, X.-H., and Fan, H.-Y. (2020). Dynamics and clinical relevance of maternal mRNA clearance during the oocyte-to-embryo transition in humans. Nat. Commun. *11*, 4917.

Sjostrom, S.K., Finn, G., Hahn, W.C., Rowitch, D.H., and Kenney, A.M. (2005). The Cdk1 complex plays a prime role in regulating N-Myc phosphorylation and turnover in neural precursors. Dev. Cell *9*, 327–338.

Soneson, C., and Robinson, M.D. (2018). Bias, robustness and scalability in single-cell differential expression analysis. Nat. Methods *15*, 255–261.

Sun, Y.H., Wang, A., Song, C., Shankar, G., Srivastava, R.K., Au, K.F., and Li, X.Z. (2021). Single-molecule long-read sequencing reveals a conserved intact long RNA profile in sperm. Nat. Commun. *12*, 1361.

Suo, L., Zhou, Y.X., Jia, L.L., Wu, H.B., Zheng, J., Lyu, Q.F., Sun, L.H., Sun, H., and Kuang, Y.P. (2018). Transcriptome profiling of human oocytes experiencing recurrent total fertilization failure. Sci. Rep. *8*, 17890.

Swain, J.E., and Pool, T.B. (2008). ART failure: oocyte contributions to unsuccessful fertilization. Hum. Reprod. Update *14*, 431–446.

Temeles, G.L., and Schultz, R.M. (1997). Transient polyadenylation of a maternal mRNA following fertilization of mouse eggs. J. Reprod. Fertil. *109*, 223–228.

Tesarík, J., Kopecný, V., Plachot, M., and Mandelbaum, J. (1988). Early morphological signs of embryonic genome expression in human preimplantation development as revealed by quantitative electron microscopy. Dev. Biol. *128*, 15–20.

Vassena, R., Boué, S., González-Roca, E., Aran, B., Auer, H., Veiga, A., and Izpisua Belmonte, J.C. (2011). Waves of early transcriptional activation and pluripotency program initiation during human preimplantation development. Development *138*, 3699–3709.

VerMilyea, M.D., Maneck, M., Yoshida, N., Blochberger, I., Suzuki, E., Suzuki, T., Spang, R., Klein, C.A., and Perry, A.C.F. (2011). Transcriptome asymmetry within mouse zygotes but not between early embryonic sister blastomeres. EMBO J *30*, 1841–1851.

Vervoorts, J., Lüscher-Firzlaff, J., and Lüscher, B. (2006). The ins and outs of MYC regulation by posttranslational mechanisms. J. Biol. Chem. *281*, 34725–34729.

Wang, B., Li, Z., Wang, C., Chen, M., Xiao, J., Wu, X., Xiao, W., Song, Y., and Wang, X. (2013). Zygotic G2/M cell cycle arrest induced by ATM/Chk1 activation and DNA repair in mouse embryos fertilized with hydrogen peroxide-treated epididymal mouse sperm. PLoS One *8*, e73987.

Wang, E.T., Sandberg, R., Luo, S., Khrebtukova, I., Zhang, L., Mayr, C., Kingsmore, S.F., Schroth, G.P., and Burge, C.B. (2008). Alternative isoform regulation in human tissue transcriptomes. Nature *456*, 470–476.

Wu, J., Xu, J., Liu, B., Yao, G., Wang, P., Lin, Z., Huang, B., Wang, X., Li, T., Shi, S., et al. (2018). Chromatin analysis in human early development reveals epigenetic transition during ZGA. Nature *557*, 256–260.

Xue, Z., Huang, K., Cai, C., Cai, L., Jiang, C.-y., Feng, Y., Liu, Z., Zeng, Q., Cheng, L., Sun, Y.E., et al. (2013). Genetic programs in human and mouse early embryos revealed by single-cell RNA sequencing. Nature *500*, 593–597.

Yamashiro, C., Sasaki, K., Yabuta, Y., Kojima, Y., Nakamura, T., Okamoto, I., Yokobayashi, S., Murase, Y., Ishikura, Y., Shirane, K., et al. (2018). Generation of human oogonia from induced pluripotent stem cells *in vitro*. Science *362*, 356–360.

Yan, L., Yang, M., Guo, H., Yang, L., Wu, J., Li, R., Liu, P., Lian, Y., Zheng, X., Yan, J., et al. (2013). Single-cell RNA-Seq profiling of human preimplantation embryos and embryonic stem cells. Nat. Struct. Mol. Biol. *20*, 1131–1139.

Yanagida, A., Spindlow, D., Nichols, J., Dattani, A., Smith, A., and Guo, G. (2021). Naive stem cell blastocyst model captures human embryo lineage segregation. Cell Stem Cell *28*, 1016–1022.e4.

Yanagimachi, R. (1994). Mammalian fertilization. In The Physiology of Reproduction, *1*, E. Knobil and J.D. Neill, eds. *(Raven Press), pp. 189–317.*

Yeh, C.D., Richardson, C.D., and Corn, J.E. (2019). Advances in genome editing through control of DNA repair pathways. Nat. Cell Biol. *21*, 1468–1478.

Yu, L., Wei, Y., Duan, J., Schmitz, D.A., Sakurai, M., Wang, L., Wang, K., Zhao, S., Hon, G.C., and Wu, J. (2021). Blastocyst-like structures generated from human pluripotent stem cells. Nature *591*, 620–626. https://doi.org/10.1038/s41586-021-03356-y.

Zheng, W., Zhou, Z., Sha, Q., Niu, X., Sun, X., Shi, J., Zhao, L., Zhang, S., Dai, J., Cai, S., et al. (2020). Homozygous mutations in *BTG4* cause zygotic cleavage failure and female infertility. Am. J. Hum. Genet. *107*, 24–33.

Zhou, D., Suzuki, T., Asami, M., and Perry, A.C.F. (2019). Caput epididymidal mouse sperm support full development. Dev. Cell *50*, 5–6.

Zhou, L.-q., and Dean, J. (2015). Reprogramming the genome to totipotency in mouse embryos. Trends Cell Biol *25*, 82–91.

CellPress
OPEN ACCESS

STAR★METHODS

KEY RESOURCES TABLE

REAGENT or RESOURCE	SOURCE	IDENTIFIER
Biological samples		
Human metaphase II (mII) oocytes	Ovation Fertility Austin, Embryology and Andrology Laboratories, Austin, TX 78731, USA	https://www.ovationfertility.com
Human bipronuclear (2PN) embryos	Ovation Fertility Austin, Embryology and Andrology Laboratories, Austin, TX 78731, USA	https://www.ovationfertility.com
Human monopronuclear (1PN) embryos	Ovation Fertility Austin, Embryology and Andrology Laboratories, Austin, TX 78731, USA	https://www.ovationfertility.com
Human tripronuclear (3PN) embryos	Ovation Fertility Austin, Embryology and Andrology Laboratories, Austin, TX 78731, USA	https://www.ovationfertility.com
Critical commercial assays		
Clontech SMARTer Total RNA-Seq Kit Pico Input (V2) system	Takara Clontech	Cat # 634412
Deposited data		
RNA-seq data	This paper	GEO: GSE157834
RNA-seq data	Leng et al., 2019	GEO: GSE133856
RNA-seq data	Xue et al., 2013	GEO: GSE44183
RNA-seq data	Wu et al., 2018	GEO: GSE101571
RNA-seq data	Sun et al., 2021	GEO: GSE137490
Experimental models: Organisms/strains		
Human (*Homo sapiens*)		N/A
Oligonucleotides		
Primers for qPCR: see Table S2	This paper	N/A
Software and algorithms		
Human GRCh38 genome and Ensembl 92 gene model using STAR (2.5.0a)	Dobin et al., 2013	N/A
Stringtie (90 1.3.6)	Pertea et al., 2015	N/A
htseq-count (0.6.1p1)	Anders et al., 2015	N/A
edgeR	Robinson et al., 2010	N/A
trim-mean of M values (TMM) normalization from the edgeR package	Robinson and Oshlack, 2010	N/A
Rtsne package		https://cran.r-project.org/web/packages/Rtsne/Rtsne.pdf
limma package	Ritchie et al., 2015	N/A
Qiagen Ingenuity Pathway Analysis (IPA) software		https://digitalinsights.qiagen.com/products/qiagen-ipa/latest-improvements/current-line/
Sashimi plots	Katz et al., 2010; Wang et al., 2008	N/A
Integrative Genome Viewer (IGV) version 2.4.19	Robinson et al., 2011	N/A

RESOURCE AVAILABILITY

Lead contact

Further information and requests for resources and reagents should be directed to the lead contact, Tony Perry (perry135@aol.com).

Materials availability

This study did not generate new unique reagents.

Data and code availability

All data are available in the main text or the supplementary materials. Source data are provided for figures. Single-cell RNA sequencing (scRNA-seq) data have been deposited into GEO with accession number GEO: GSE157834.

EXPERIMENTAL MODEL AND SUBJECT DETAILS

Human oocyte and embryo sample collection

Human oocytes and single monopronuclear (1PN), bipronuclear (2PN) and tripronuclear (3PN) one-cell embryos were supplied anonymously subject to informed consent for use in research by couples who had finished family building or decided for other reasons to discontinue fertility treatment. Consents strictly adhered to guidelines of the Ethics Committee of the American Society for Reproductive Medicine. Embryos were cryopreserved and lysed on site at *Ovation Fertility* before being anonymized and shipped for analysis.

METHOD DETAILS

Human metaphase II oocytes and one-cell embryos (zygotes)

Patients underwent ovarian stimulation according to guidelines of each clinic, where protocols included agonist luteal phase and antagonist suppression. On the day of retrieval (day 0), mature, metaphase II (mII) oocytes were either cryopreserved by a slow freeze method using propanediol (PROH) (Gook et al., 1993) 3-6 h post-collection, or used to produce embryos by *in vitro* fertilization (IVF) or ICSI. One-cell embryos used here were morphologically assessed for pronuclear number 19-23 h post-fertilization, cryopreserved 1-5 h later using dimethylsulfoxide (Camus et al., 1989) and stored under liquid nitrogen. Some sibling embryos of morphologically normal bipronuclear (2PN) one-cell embryos (i.e., each containing two pronuclei) gave rise to children. When required, cryopreserved oocytes and one-cell embryos were thawed by rapid warming using a Vit-Warm Kit (FUJI Irvine Scientific, USA) according to the recommended protocol, and viability confirmed. All mII oocytes and one-cell embryos were washed in protein-free multi-purpose handling medium (FUJI Irvine Scientific, USA) and each placed in a 0.2 ml PCR tube containing 0.8 μl 1x single-cell lysis buffer supplemented with RNase inhibitor (Takara Clontech, USA). Oocyte and one-cell embryo (2PN; monopronuclear, 1PN; tripronuclear, 3PN) donor groups did not overlap; mII oocytes and one-cell embryos came from different individuals (Table S3). For scRNA-seq, there were seven mII oocyte donors (six were aged 22.5, 24.5, 25, <30, 27 and 31 years); six were Caucasian and one African American/Hispanic. There were six 2PN one-cell embryo donor couples; for two, male and female ages were respectively 36 and 38, and 40 and 50 (data are unavailable for the other couples) and five of the couples were Caucasian, with one Asian couple.

Single-cell RNA sequencing (scRNA-seq) of oocytes and one-cell embryos

RNA sequencing libraries from 14 mII oocytes and 13 bipronuclear (2PN) one-cell embryos (Table S3) were prepared using the Clontech SMARTer Total RNA-Seq Kit Pico Input (V2) system (Takara Clontech). Briefly, total RNA was liberated by lysis of single oocytes or one-embryos in 0.8 μl lysis buffer supplemented with RNase inhibitor (both from Takara Clontech). The RNA was incubated with SMART Pico N6 primers at 72°C for 3 min and then subjected to first-strand synthesis with SMARTScribe reverse transcription using a Pico v2 SMART adapter (template-switching oligo, TSO). After first strand synthesis, cDNA amplification was performed using SeqAmp DNA polymerase with Illumina barcoded adapters for 5 cycles of 15 sec at 98°C; 15 sec at 55°C; 15 sec at 68°C, followed by final extension for 2 min at 68°C. Ribosomal cDNA was removed using the ZapR v2 and R-Probes v2, after which there was a second round of cDNA amplification with SeqAmp DNA Polymerase for 15 cycles of 15 sec at 98°C, 15 sec at 55°C, and 30 sec at 68°C, to generate the final sequencing libraries. Libraries were analyzed using High-sensitivity D1000 ScreenTape and Agilent TapeStation 4200 (Agilent, USA). All samples were subjected to the same amplification protocol and produced indistinguishable final library yields, with mean±SD respectively of 54.9±25.3 nM and 50.7±9.7 nM for oocytes and one-cell embryos; p=0.595). For next-generation sequencing, libraries were combined at equimolar concentrations before loading onto an Illumina NovaSeq 6000 instrument (Illumina, USA) for paired-end 100 (PE100) sequencing to generate an average of ~88.2 million raw read pairs per sample.

Ratiometric real-time-PCR (qPCR)

For qPCR, total RNA was liberated by lysis of single oocytes or one-embryos in 0.8 μl lysis buffer supplemented with RNase inhibitor (Takara Clontech). Synthesis of cDNA employed a method modified from the Clontech SMARTer protocol using a total RNA-Seq Kit Pico Input (V2). In brief, total RNA was incubated with SMART Pico Oligos mix v2 at 72°C for 3 min and immediately chilled on ice for 2 min. Samples were then subjected to first-strand synthesis with SMARTSCribe RT (Takara Clontech). First step cDNA amplification was performed using SeqAmp DNA Polymerase, skipping the addition of Illumina barcode adapters and incubating for 1 min at 94°C, followed by 5 cycles of (15 sec at 98°C; 15 sec at 55°C; 15 sec at 68°C) and then for 2 min at 68°C. Second step amplification was performed with SeqAmp DNA Polymerase and PCR2 Primer 2v2, incubating for 1 min at 94°C, followed by 16 cycles of (15 sec at 98°C; 15 sec at 55°C; 30 sec at 68°C) and cDNA stored at -20°C until required. qPCR reactions were performed in a QuantStudio 7 (Thermo Fisher Scientific, UK) or ABI 7500 Real Time PCR System (Applied Biosystems, CA) in reactions (20 μl) containing

1-2 μl template cDNA, forward and reverse primers (100 nM each) and 12.5 μl of Power SYBR (ABI), using standard parameters. Data for each target were obtained from n=3 or 4 biological replicates (*i.e.,* independent single cells) collected on at least two days, and included technical replicates of each biological replicate. Primer sequences are given in Table S2. Primer sets (Sigma-Merck) were non-dimerizing under the conditions employed. Reactions lacking input cDNA were used to verify absence of contamination in cocktail components. Steady state transcript levels were normalized with respect to internal reference, RNA18s5, or in most cases, *H3F3A*. *H3F3A* gave mean cycle threshold (Ct) values (±s.e.m.) of 24.51±0.30 for mII oocytes (n=45 replicates over all, n=5 biological replicates) and 24.56±0.33 for 2PN one-cell embryos (n=51 replicates over all, n=8 biological replicates); p=0.829 for mII oocytes *vs* embryos.

QUANTIFICATION AND STATISTICAL ANALYSIS

Bioinformatic analysis

Raw read pairs were mapped onto the Human GRCh38 genome and Ensembl 92 gene model using STAR (2.5.0a) (Dobin et al. 2013), employing the following parameters: (–outFilterScoreMinOverLread 0.3 –outFilterMatchNminOverLread 0.3 –outSAMstrandField intronMotif –outSAMtype BAM SortedByCoordinate). Post-alignment reads (BAMs) were further processed by Stringtie (1.3.6) (Pertea et al., 2015) to remodel the transcriptome using default parameters and the Ensembl 92 gene model as base reference. Once the reassembly was complete, a gene-level count was performed using htseq-count (0.6.1p1) (Anders et al., 2015). For transcript-level analysis, Stringtie was used to generate estimated counts for all detected transcripts.

Gene- and transcript-level count tables were imported into edgeR (Robinson et al., 2010) for downstream differential gene expression analysis. For gene-level analysis, genes expressed at low levels (<1 count per million, >13 samples) were filtered, retaining a final total of 16,625 genes. Due to minimal differences detected in the library yields (see above), samples were subjected to trim-mean of M values (TMM) normalization from the edgeR package (for details, see Robinson and Oshlack, 2010), a common normalization method where a weighted trimmed mean of the log expression ratios is used to normalize sequencing depth. This has been shown to be among the most robust methods for RNA-sequencing differential expression analysis, including single-cell studies where global gene expression differences are large (Evans et al., 2018; Soneson and Robinson, 2018). A subtle normalization factor of 1.00±0.07 (mean±SD) was applied. Normalized gene abundance was listed as read counts per million of mapped reads (CPM) and determined using the formula:

$$\text{CPM} = \text{raw count}/\text{sample library size} * \text{normalization factor}$$

To generate a visual overview of transcriptome profiles, we employed the Rtsne package (https://cran.r-project.org/web/packages/Rtsne/Rtsne.pdf) to perform dimensionality reduction via the principal component analysis (PCA, ndims=50) coupled with t-distributed stochastic neighbor embedding (t-SNE), using the normalized abundance (CPM) of all genes as input with the default parameters. The advantage of using t-SNE over PCA is that it provides superior resolution with which to differentiate samples in fewer dimensions (two projections were used here) and it is commonly used in single-cell RNA sequencing studies. Two mII oocytes were excluded as outliers, and one 2PN one-cell embryo failed to amplify and had to be abandoned (Table S3). The remaining 24 samples (n=12 each oocytes and one-cell embryos) were re-normalized (mean normalization factor ±SD: 1.03±0.02 for mII oocytes and 0.97±0.03 for one-cell embryos) to give a final average of 66.3±9.5 (±SD) million usable read pairs. t-SNE was also re-performed using the same parameters. For differential gene expression, a generalized linear model (GLM) was applied to determine the common, trend and gene-wise dispersions, and likelihood-ratio tests were employed to detect differential gene expression. Genes with a false discovery rate (FDR) of <0.1 were considered differentially expressed.

For transcript-level analysis, transcripts from the 24 samples with low expression were first removed and estimated transcript level counts TMM-normalized (normalization factor = 1.00±0.05) and CPM determined by limma-voom (Law et al., 2014). The total number of transcripts that remained in the analysis was 42,230. We then calculated a gene-variability statistic to adjust for the mean-variance relationship using limma-voom and differential expression was determined using empirical Bayesian t-test (eBayes) from the limma package (Ritchie et al., 2015). Transcripts with an FDR <0.1 were considered differentially expressed.

Pathway and upstream regulator analyes were performed using Qiagen Ingenuity Pathway Analysis (IPA) software using HGNC gene symbols and a raw cut-off of p<0.05. Loci encompassing multiple genes were split in the analysis. Sashimi plots (Katz et al., 2010; Wang et al., 2008) were generated using all mapped reads accumulated from oocytes and one-cell embryos using Integrative Genome Viewer (IGV) version 2.4.19 (Robinson et al., 2011).

For bioinformatic analysis of data from Leng et al. (2019), 65 raw fastq files for individual blastomeres of biparental two-, four- and eight-cell embryo were downloaded via the Gene Expression Omnibus (accession number GSE133854) using the fastq-dump command from the SRA toolkit, with the –split-files argument to split reads 1 & 2. The fastq files were then mapped onto the Human GRCh38 genome, and gene abundance (Ensembl V97) counted using standard STAR 2.5.0a, as described above. The average usable read pair counts (uniquely mapped read pairs to gene) per sample was 24.47 ± 1.2 (s.e.m.) million. Count data were imported into edgeR (3.32.1) and normalized via TMM. Samples were quality checked using the plotMDS() function and samples SRR9645989, SRR9645990, SRR9645991, SRR9645992, SRR9645993, SRR9645994, SRR9645995, SRR9645996 and SRR9645997 were removed, leaving a total of 56 samples. Genes with low expression (<1 CPM in $\geq$ 46 samples) were removed and counts re-normalized with an average normalization factor of 1.012 ± 0.020. Differential gene expression across all stages was determined by fitting a generalized log linear model followed by likelihood ratio test with a false discovery rate (FDR) cutoff of <0.1; 11,083 (out of 16,810)

genes were detected as being differentially expressed. The intersection of the differentially expressed genes in this dataset and the top 200 up-regulated genes (ranked by FDR) from our mII vs one-cell embryo dataset were extracted and we found the majority of the genes (153/200; 76.5%) were differentially expressed in both datasets. A heatmap of the 153 genes was drawn using the pHeatmap package (1.0.12), employing z-scoring to reveal clusters of gene expression trajectories.

Statistical analysis

Each experiment used $n \geq 3$ experimental samples, as indicated in the text and figure legends, and are presented as mean ± s.e.m. Statistical differences between pairs of qPCR datasets were analyzed by two-tailed unpaired t-tests. Values of $p < 0.05$ were considered statistically significant unless stated otherwise.

Cell Stem Cell, Volume *29*

Supplemental Information

Human embryonic genome activation
initiates at the one-cell stage

Maki Asami, Brian Y.H. Lam, Marcella K. Ma, Kara Rainbow, Stefanie Braun, Matthew D. VerMilyea, Giles S.H. Yeo, and Anthony C.F. Perry

SUPPLEMENTAL INFORMATION

Human embryonic genome activation initiates at the one-cell stage

Maki Asami, Brian Y. H. Lam, Marcella K. Ma, Kara Rainbow, Stefanie Braun, Matthew D. VerMilyea, Giles S. H. Yeo, Anthony C. F. Perry

This file includes:

Legends for Figures S1 and S2

Figures S1 and S2

Tables S2 and S3

Other Supplemental Information for this manuscript includes:

Table S1 (.xls)

Figure S1. Human one-cell embryo expression profiles and relationships

(A) Volcano plot showing genes exhibiting upregulated (up), downregulated (down) and non-changing (nc) expression in one-cell embryos from scRNA-seq. Top upregulated (blue) and down regulated (pink) genes are indicated.

(B) Upregulated gene number (FDR<0.1) from scRNA-seq of one-cell embryos at each log2FC (upper bound indicated for each bin).

(C) Ingenuity Pathway Analysis of down-regulated gene networks (FDR<0.10) in human one-cell embryos.

(D) Venn Diagrams showing upregulated gene overlap between previous datasets from human one-cell (1C) embryo RNA-seq and RNA-seq for two-cell (2C), four-cell (4C) and eight-cell (8C) embryos (L, Leng et al., 2019; X, Xue et al., 2013; W, Wu et al., 2018). Values in parentheses show percentages of the total in one-cell embryos. Applied parameters are provided in references or applied to datasets as follows: L-1C, L-2C, L-4C, L-8C (Leng et al., 2019); X-1C (p_{adj}<0.06, log2FC>2), X-2C (p_{adj}<0.6, log2FC>2), X-4C (p_{adj}<1, log2FC>2), X-8C (p_{adj}<0.06, log2FC>2); W-2C (log2FC>1), W-8C (log2FC>1).

(E) Venn Diagrams showing upregulated gene overlap between human one-cell embryo scRNA-seq obtained here (FDR<0.1, logFC>0.5) and data previously reported for one-cell (1C), two-cell (2C), four-cell (4C) and eight-cell (8C) embryos (L, Leng et al., 2019; X, Xue et al., 2013; W, Wu et al., 2018) or sperm intact RNA (sperm) (Sun et al., 2021) (FDR<0.1), showing (in parentheses) percentage of the total in one-cell embryos. Comparisons were of annotated genes using parameters of (D).

(F) Heatmap indicating expression trajectories of 153 of the top 200 upregulated DEGs from one-cell embryos determined by scRNA-seq in this work (FDR<0.1), relative to expression in single blastomeres from two-, four- and eight-cell embryos (Leng et al., 2019). Genes were sorted into three clusters (I, II, and III) as indicated.

See also Figure 1 and Table S1.

Figure S2. Differential gene expression networks in human one-cell embryos

(A) Histograms derived from scRNA-seq showing endogenous retrovirus (*hERV*) loci upregulated in human one-cell embryos. Locus coordinates are shown on the x-axis.

(B) Histograms derived from scRNA-seq showing endogenous retrovirus (*hERV*) loci down-regulated in human one-cell embryos. Locus coordinates are given on the x-axis.

(C) Pie chart showing functional classes of upregulated DEGs (FDR<0.10) related to molecular mechanisms of cancer (Ingenuity).

(D) Upstream transcription activators predicted by Ingenuity Pathway Analysis (IPA) of upregulated DEGs in human one-cell embryos (FDR<0.10).

(E-H) Upstream transcription regulators and associated networks inferred by IPA of upregulated gene networks (FDR<0.10) in human one-cell embryos for RABL6 (E), FOXM1 (F), YAP1 (G) and TP53 (H).

(I) Genes in the E2F4 cluster identified by IPA.

See also Figure 2 and Table S1.

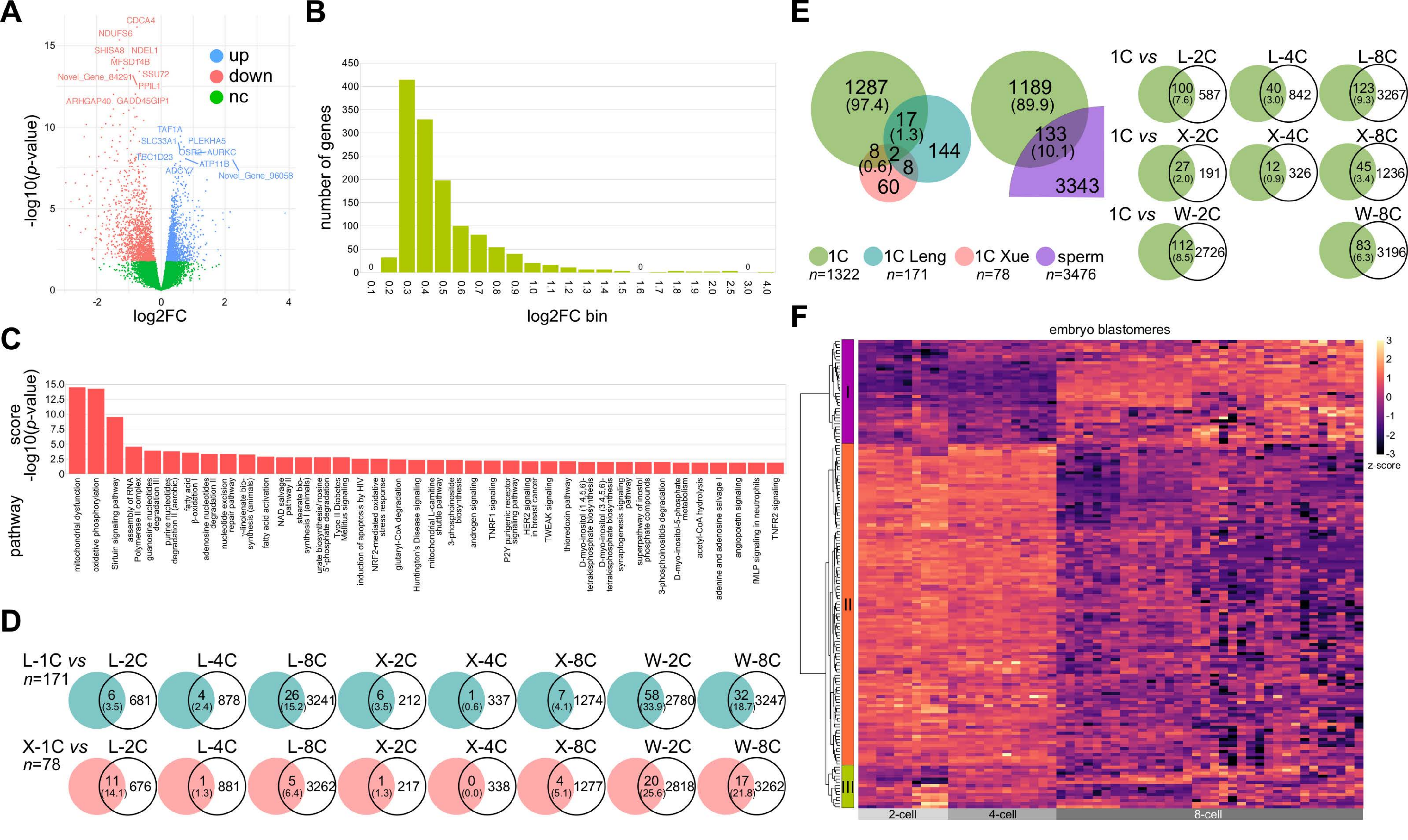

Figure S1
Asami et al.

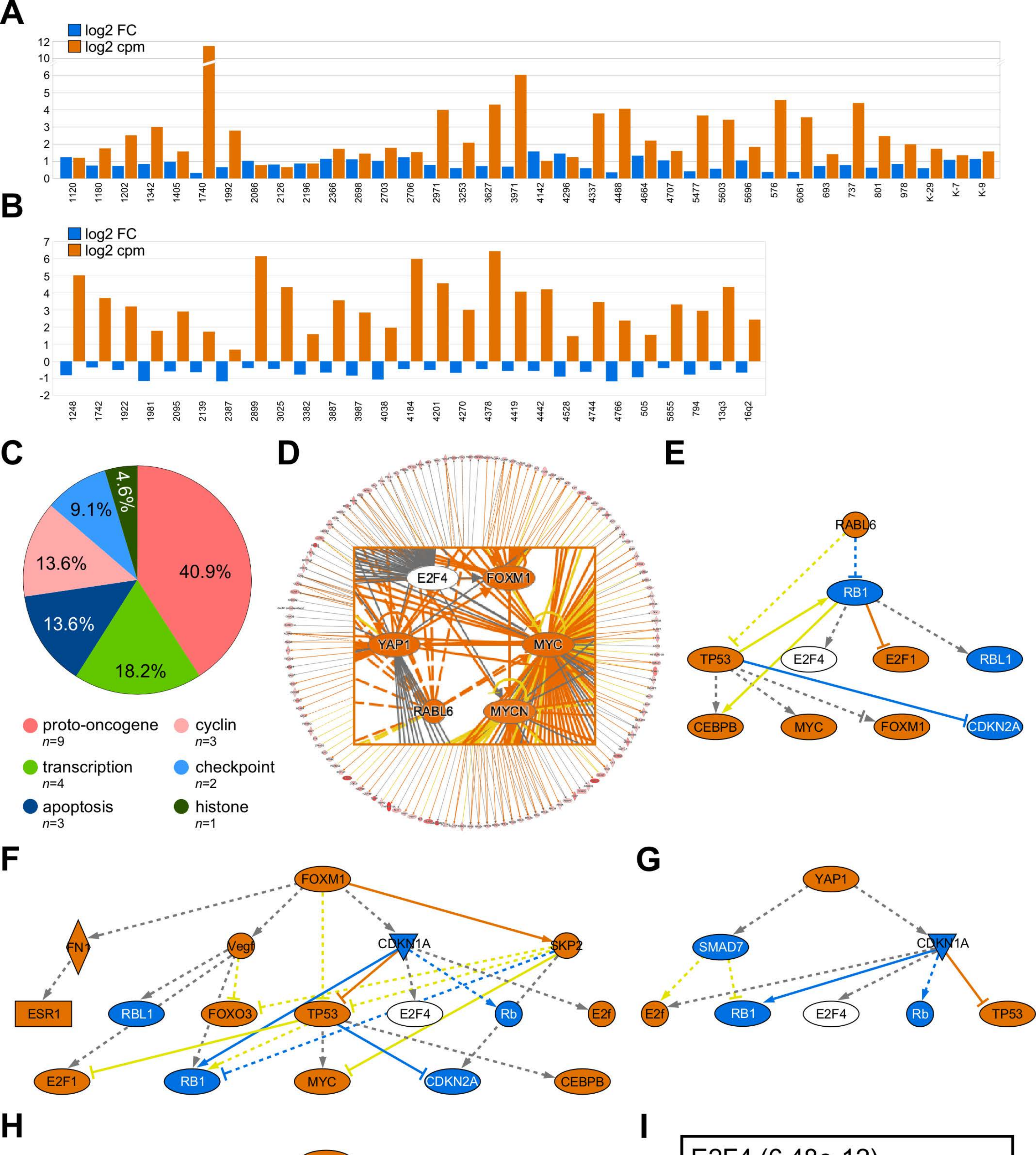

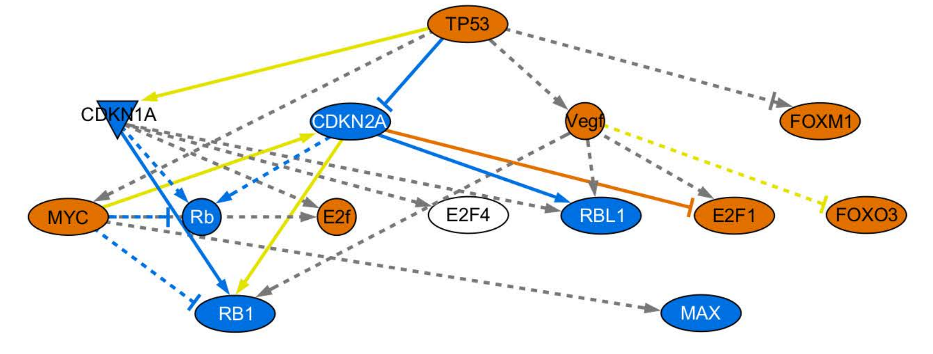

I

E2F4 (6.48e-12)		
ANLN	H2AC6	POLA1
AURKB	H2AX	PRC1
BARD1	HMGB3	PRKDC
C5orf22	HMMR	PTTG1
CALM1	KIF4A	RAD54L
CCDC77	MAD2L1	RBL2
CCNE1	MCM10	TMPO
CCNE2	MCM6	TOP2A
CDK1	NEK2	TP53INP1
DDX11	NPM1	TPX2
DROSHA	ORC1	TRMT13
FDFT1	PCNA	ZNF267

Figure S2
Asami et al.

Table S2 PCR Primers used in this work, related to Figures 1 and 2

Sequence	Forward (5'→3')	Reverse (5'→3')
H3F3A	ACAAAAGCCGCTCGCAAGAGTG	TTTCTCGCACCAGACGCTGGAA
CDH15	AGCGTATCCGAGAACCACAAGC	CTGGATGCTGTAGATGACGCTG
TAF1A	TCTGTGCTCAGTGGTGCAGGAA	TGCCATTGGTGCTTCAGCAGAG
ZCCHC8	TTTCCAGCAGCGATACCACGCA	GGAAGACTCTTGTCTGTCACACC
AIF1L	CCTTCCAGAAAAGCTCACAGCC	CTTCATCTCCAGGTGGGTCTTG
TXNIP	CAGCAGTGCAAACAGACTTCGG	CTGAGGAAGCTCAAAGCCGAAC
TIGD5	GTGCTGTTCCTGTCCAAAGGCA	AGGACACAGCCAGTCGCAGCA
CMTR2	CTGACCACTCTTGGAAACGGTG	GACTTCGGAGTTTCCTGCCTTG
RIPK4	CTGAAACCGAGGACCTGTGTGA	GCTGTAGTCGTTATCGAAGGTGG
OPRM1	CGTCAGAACACTAGAGACCACC	CTTGGTGAAGGTCGGAATGGCA
RAP2C	GAGCAGTTTGCCTCCATGAGAG	CCTACTAGGATTAGTGGGACTTTT
PIAS3	TGGTGATGAGTTTCCGGGT	GGAAAGCGTCGTCGGTAA
UGDH	TGTGATGGTGCCCATGCTGTTG	GTCCATCGAAGATAAAGGCTGGC
NFKBIA	TCCACTCCATCCTGAAGGCTAC	CAAGGACACCAAAAGCTCCACG
CCNE1	TGTGTCCTGGATGTTGACTGCC	CTCTATGTCGCACCACTGATACC

Table S3 Human embryos used in this work

#	Patient	Sample	Analysis	Ethnicity	Age (yr)	Yield (nM)	Useable reads	Normalization factor (TMM)	Genes detected (≥1cpm)
1	1	2PN	RNAseq	Asian	nd	61.7	68635512	0.9692079	15709
2	1	2PN	RNAseq	Asian	nd	60.8	65181145	0.9699501	15601
3	1	2PN	RNAseq	Asian	nd	48.8	88623885	0.9031541	15732
4	2	2PN	RNAseq	Caucasian	nd	31.2	46084528	1.001886	15726
5	2	2PN	RNAseq	Caucasian	nd	3.1	Amplification failure	nd	nd
6	3	2PN	RNAseq	Caucasian	nd	54.8	67065772	0.981549	15763
7	4	2PN	RNAseq	Caucasian	nd	52.9	76729549	0.957278	15630
8	4	2PN	RNAseq	Caucasian	nd	46.2	76189001	0.944969	15762
9	4	2PN	RNAseq	Caucasian	nd	51.6	73264323	0.9851035	15918
10	5	2PN	RNAseq	Caucasian	nd	43.7	75180066	0.9368282	15767
11	5	2PN	RNAseq	Caucasian	nd	46.6	73222174	0.9957537	15831
12	6	2PN	RNAseq	Caucasian	nd	67.1	66404656	0.9754091	15753
13	6	2PN	RNAseq	Caucasian	nd	43.5	58715420	0.9919022	15602
14	7	mII	RNAseq	Caucasian	nd	46.7	72657324	0.9876494	15585
15	7	mII	RNAseq	Caucasian	nd	15.4	Excluded outlier	nd	nd
16	8	mII	RNAseq	Caucasian	nd	66.1	65324304	1.0415315	15825
17	8	mII	RNAseq	Caucasian	nd	52.2	72466449	1.0608596	15885
18	9	mII	RNAseq	Caucasian	<30	72.5	Excluded outlier	nd	nd
19	9	mII	RNAseq	Caucasian	<30	25.2	54705074	1.0171751	15455
20	10	mII	RNAseq	Caucasian	24.5	68.2	51126979	1.0306027	15860
21	10	mII	RNAseq	Caucasian	24.5	57.8	55244789	1.026353	15818
22	11	mII	RNAseq	African American/Hispanic	31	94.2	67438079	1.0240237	15812
23	11	mII	RNAseq	African American/Hispanic	31	97.4	61061212	1.0471581	15985
24	12	mII	RNAseq	Caucasian	22.5	46.3	69363653	1.0382324	15837

25	12	mII	RNAseq	Caucasian	22.5	70.1	65550194	1.0569761	15920
26	13	mII	RNAseq	Caucasian	25	21.1	67377209	1.0439114	15942
27	13	mII	RNAseq	Caucasian	25	35.8	54760799	1.0324617	15977
28	7	mII	qPCR	Caucasian	nd				
29	7	mII	qPCR	Caucasian	nd				
30	11	mII	qPCR	African American/Hispanic	31				
31	8	mII	qPCR	Caucasian	nd				
32	10	mII	qPCR	Caucasian	24.5				
33	1	2PN	qPCR	Asian	nd				
34	1	2PN	qPCR	Asian	nd				
35	1	2PN	qPCR	Asian	nd				
36	1	2PN	qPCR	Asian	nd				
37	1	2PN	qPCR	Asian	nd				
38	5	2PN	qPCR	Caucasian	nd				
39	14	2PN	qPCR	Unknown	nd				
40	15	3PN	qPCR	Unknown	nd				
41	16	3PN	qPCR	Unknown	nd				
42	3	3PN	qPCR	Caucasian	nd				
43	17	1PN	qPCR	Unknown	nd				
44	15	1PN	qPCR	Unknown	nd				
45	18	1PN	qPCR	Unknown	nd				

Abbreviations: 1PN, 2PN, 3PN, respectively monopronuclear, bipronuclear and tripronuclear human one-cell embryos; nd, no data; TMM, trim-mean of M-values; cpm, counts per million.
